# Supplementary material for: Single-component color-tunable circularly polarized organic afterglow through chiral clusterization
Source: Nat Commun. 2022 Jan 20;13:429. doi: 10.1038/s41467-022-28070-9 (PMC8776763; doi:10.1038/s41467-022-28070-9)
Supplement: Supplementary file 1 — Supplementary Information [file 41467_2022_28070_MOESM1_ESM.pdf]

Supplementary Information

Single-Component Color-Tunable Circularly Polarized Organic Afterglow through Chiral Clusterization

Hui Li[1], Jie Gu[1], Zijie Wang[1], Juan Wang[1], Fei He[1], Ping Li[1], Ye Tao[1*], Huanhuan Li[1], Gaozhan Xie[1], Wei Huang[1, 2*], Chao Zheng[1] and Runfeng Chen[1*]

Ms. H. Li, Ms. J. Gu, Ms. Z. J. Wang, Ms. J. Wang, Mr. F. He, Dr. P. Li, Prof. Y. Tao, Dr. H. H. Li, Dr. G. Z. Xie, Prof. W. Huang, Dr. C. Zheng and Prof. R. F. Chen

[1]State Key Laboratory of Organic Electronics and Information Displays & Institute of Advanced Materials (IAM), Nanjing University of Posts & Telecommunications, 9 Wenyuan Road, Nanjing 210023, China.

E-mail: iamytao@njupt.edu.cn and iamrfchen@njupt.edu.cn;

Prof. W. Huang

[2]Frontiers Science Center for Flexible Electronics (FSCFE), MIIT Key Laboratory of Flexible Electronics (KLoFE), Shaanxi Key Laboratory of Flexible Electronics, Xi'an Key Laboratory of Flexible Electronics, Xi'an Key Laboratory of Biomedical Materials & Engineering, Xi'an Institute of Flexible Electronics, Institute of Flexible Electronics (IFE), Northwestern Polytechnical University, Xi'an 710072, Shanxi, China.

E-mail: provost@nwpu.edu.cn.

Content

1. Synthesis and characterization S3

2. Thermal properties S12

3. Photophysical investigations S13

4. Single crystals analysis S25

5. Theoretical calculations S28

6. Design rationale verification of chiral clusterization strategy S40

1. Synthesis and characterization

Materials: All reagents, unless otherwise specified, were purchased from Aldrich, Acros or Alfa Aesar, and used without further purification. D/L-serine were purchased from Daicel Chiral Technologies (China) Co., LTD. Manipulations involving air-sensitive reagents were performed in an atmosphere of dry argon (Ar).

Instruments: ^{1}H and ^{13}C-nuclear magnetic resonance (NMR) spectra were recorded on Bruker Ultra Shield Plus 400 MHz instruments with DMSO-d_6 as the solvents and tetramethylsilane (TMS) as the internal standard. High resolution mass spectra (HRMS) were collected by a LCT Premier XE (Waters) HRMS spectrometry. Matrix-assisted laser desorption/ionization time of flight mass spectrometer (MALDI-TOF MS) was performed on a Buker Daltonics flex Analysis. Melting points (m.p.) were determined using a SGW X-4 micro-melting point apparatus.

Synthesis of *trans*-(1*R*, 2*R*)-cyclohexanedi (3-aminocarbonylpropionic) acid ((R, R)-DAACH)

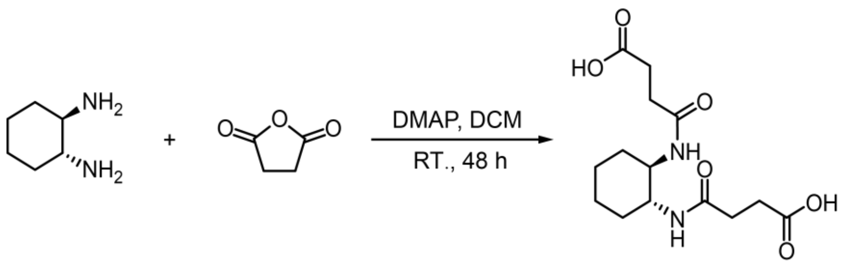

Trans-(1*R*, 2*R*)-diamidocyclohexane (1.00 g, 8.76 mmol, ≥ 99% purity) and 4-dimethylaminopyridine (20 mg, 0.18 mmol, 98% purity) were dissolved in 50 mL of dichloromethane (99.5% purity) under Ar atmosphere at room temperature. Succinic anhydride (1.84 g, 18.4 mmol, 99% purity) was dissolved in another 50 mL of dichloromethane and added slowly to the reaction mixture. The reaction was allowed to stir for 48 h at room temperature. White solid formed in the solution over the reaction course was collected by filtration, and then washed with dichloromethane several times to remove the raw materials to obtain the crude product[1]. The crude product was recrystallized by slow evaporation of a mixed deionized water and ethanol solution to achieve the purified product. Yield: 2.25 g of white powder (82%). m.p.: 198°C. ^{1}H NMR (400 MHz, DMSO-d_6, ppm): δ 12.09 (s, 2H), 7.60-7.58 (d, J= 8.0 Hz, 2H), 3.46 (s, 2H), 2.39-2.36 (m, 4H), 2.26-2.22 (m, 4H), 1.74-1.63 (m, 4H), 1.23-1.17 (t, J= 12.1 Hz, 4H);

^{13}C NMR (100 MHz, DMSO-d_6, ppm): δ 174.28, 171.22, 52.59, 32.22, 30.65, 29.74, 24.86. HRMS (EI): m/z calcd. for $C_{14}H_{23}N_2O_6$, 315.1556 $[M+H]^+$; found: 315.1551.

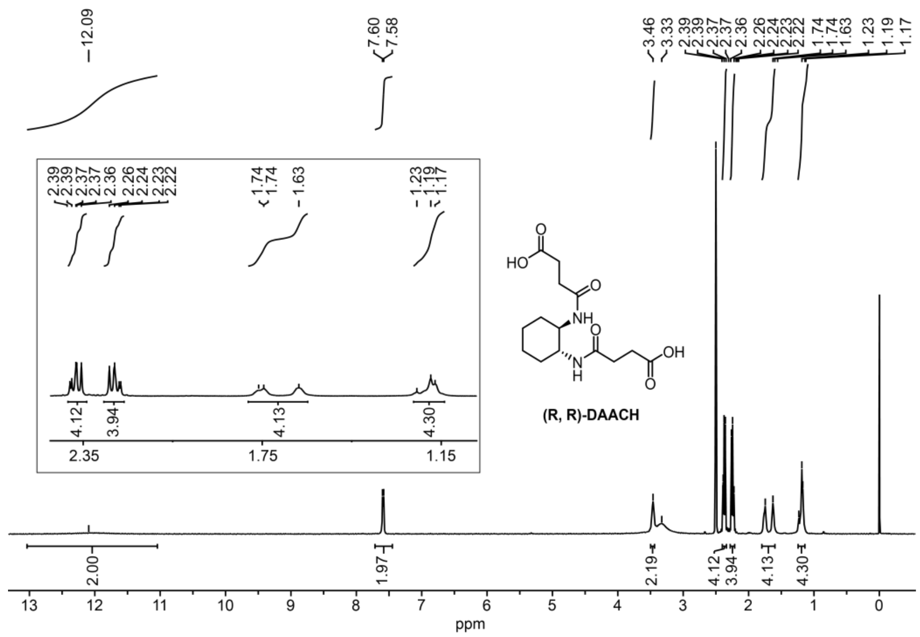

Supplementary Figure 1. ^{1}H NMR spectrum of **(R, R)-DAACH**.

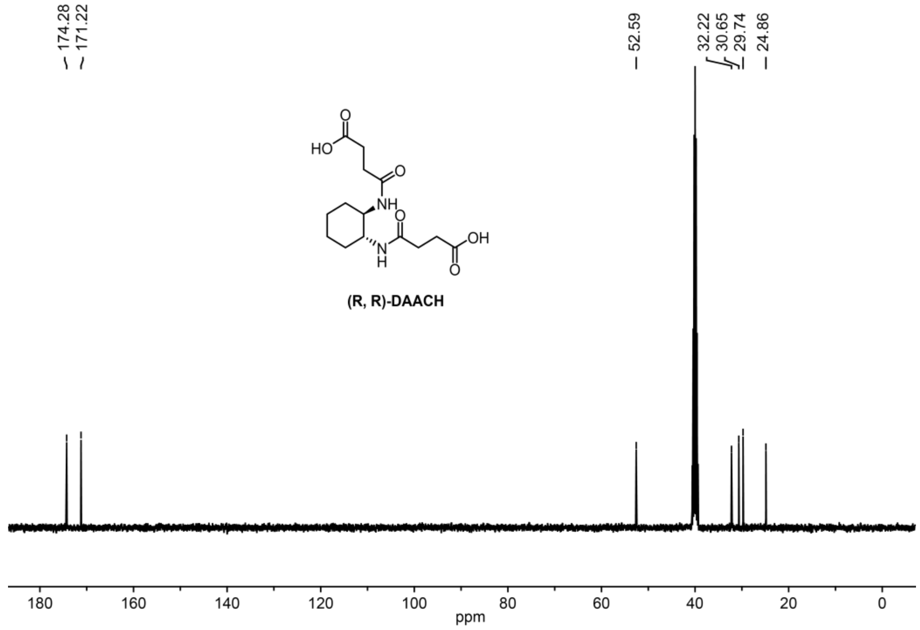

Supplementary Figure 2. ^{13}C NMR spectrum of **(R, R)-DAACH**.

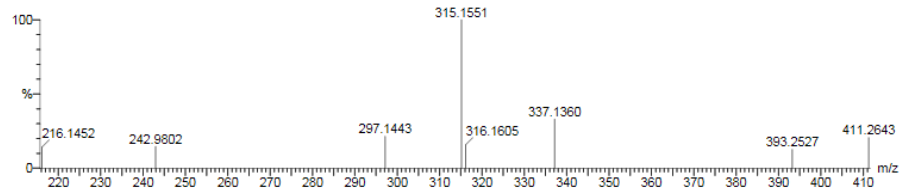

Minimum:				-1.5			
Maximum:		5.0	10.0	50.0			
Mass	Calc. Mass	mDa	PPM	DBE	i-FIT	i-FIT (Norm)	Formula
315.1551	315.1556	-0.5	-1.6	4.5	24.7	0.0	C14 H23 N2 O6

Supplementary Figure 3. HRMS of **(R, R)-DAACH**.

Synthesis of *trans*-(1*S*, 2*S*)-cyclohexanedi (3-aminocarbonylpropionic) acid ((S, S)-DAACH)

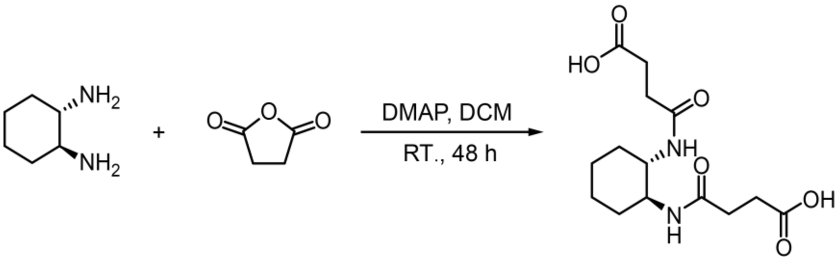

(S, S)-DAACH was prepared under the identical synthetic conditions described in the preparation of **(R, R)-DAACH** using *trans*-(1*S*, 2*S*)-diamidocyclohexane (1.00 g, 8.76 mmol, ≥ 99% purity), 4-dimethylaminopyridine (20 mg, 0.18 mmol) and succinic anhydride (1.84 g, 18.4 mmol). Yield: 2.22 g of white powder (81%). m.p.: 200°C. ^{1}H NMR (400 MHz, DMSO-d_6, ppm): δ 12.06 (s, 2H), 7.60-7.58 (d, J= 8.0 Hz, 2H), 3.46 (s, 2H), 2.40-2.36 (m, 4H), 2.26-2.22 (m, 4H), 1.75-1.63 (m, 4H), 1.23-1.17 (t, J= 12.1 Hz, 4H); ^{13}C NMR (100 MHz, DMSO-d_6, ppm): δ 174.31, 171.27, 52.60, 32.22, 30.71, 29.82, 24.86. HRMS (EI): m/z calcd. for $C_{14}H_{22}N_2O_6Na$, 337.1376 $[M+Na]^+$; found: 337.1379.

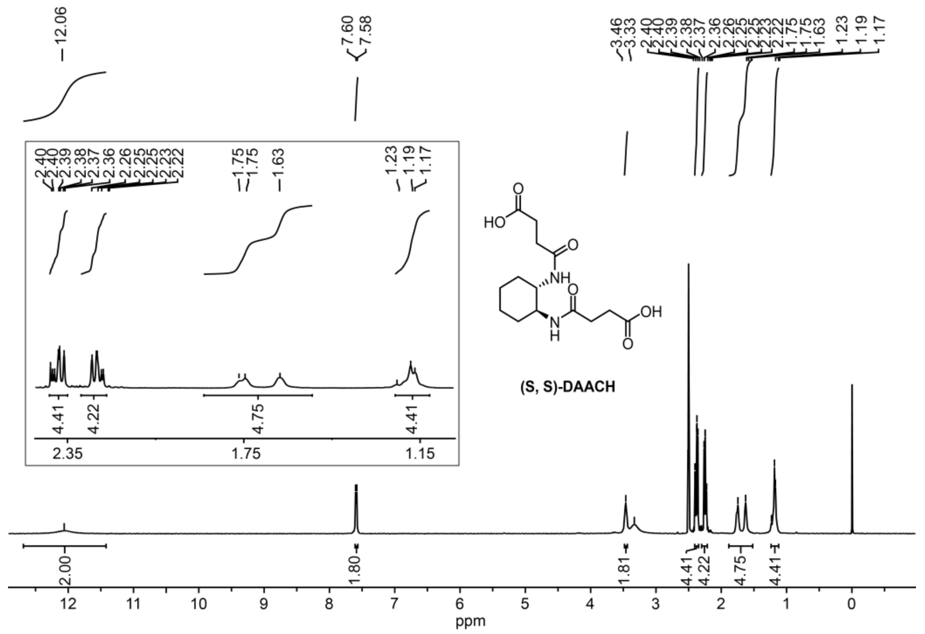

Supplementary Figure 4. ^{1}H NMR spectrum of **(S, S)-DAACH**.

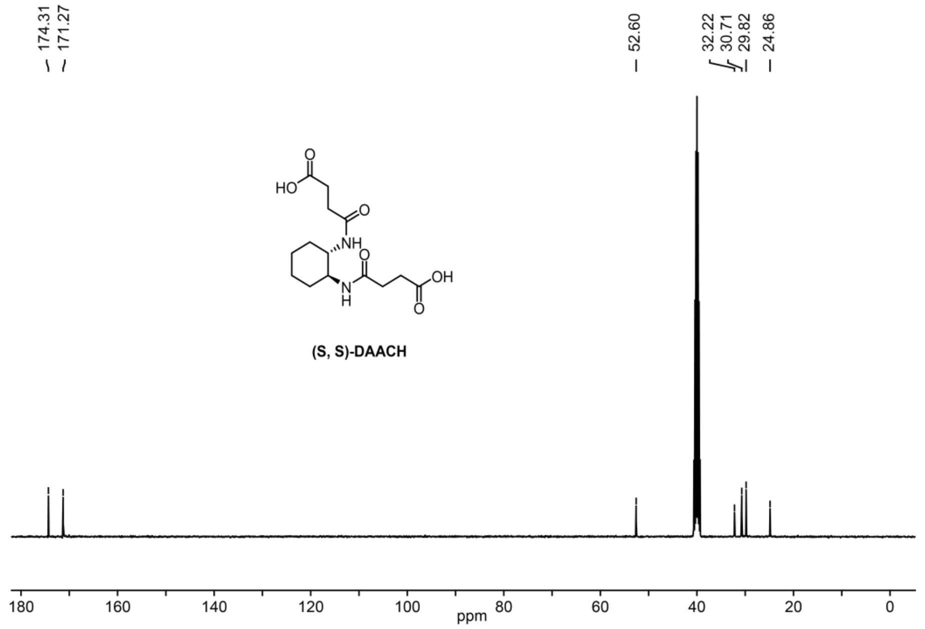

Supplementary Figure 5. ^{13}C NMR spectrum of **(S, S)-DAACH**.

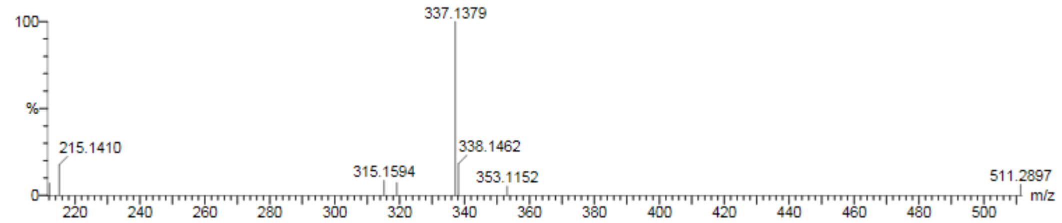

Minimum:				-1.5			
Maximum:		5.0	10.0	50.0			
Mass	Calc. Mass	mDa	PPM	DBE	i-FIT	i-FIT (Norm)	Formula
337.1379	337.1376	0.3	0.9	4.5	34.8	0.0	C14 H22 N2 O6 Na

Supplementary Figure 6. HRMS of **(S, S)-DAACH**.

Synthesis of *trans*-(1*R*, 2*R*)-cyclohexanedi (3-aminocarbonylbutanoic) acid ((R, R)-DAPCH)

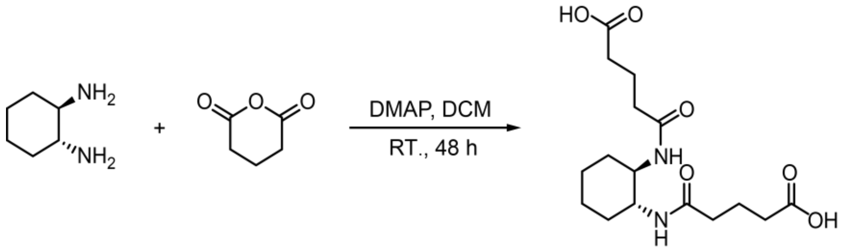

(R, R)-DAPCH was prepared under the identical synthetic conditions described in the preparation of **(R, R)-DAACH** using *trans*-(1*R*, 2*R*)-diamidocyclohexane (1.00 g, 8.76 mmol), 4-dimethylaminopyridine (20 mg, 0.18 mmol) and glutaric anhydride (2.09 g, 18.4 mmol, 99% purity). Yield: 2.55 g of white powder (85%). m.p.: 191°C. ^{1}H NMR (400 MHz, DMSO-d_6, ppm): δ 12.05 (s, 2H), 7.60-7.58 (d, J= 7.9 Hz, 2H), 3.46 (s, 2H), 2.19-2.14 (m, 4H), 2.04-2.01 (t, J = 7.4 Hz, 4H), 1.76-1.63 (m, 8H), 1.23-1.17 (t, J = 12.9 Hz, 4H); ^{13}C NMR (100 MHz, DMSO-d_6, ppm): δ 174.69, 171.80, 52.25, 35.11, 33.44, 32.46, 24.93, 21.23. MALDI-TOF: m/z calcd for $C_{16}H_{27}N_2O_6$: 343.186 $[M+H]^+$; found: 343.009.

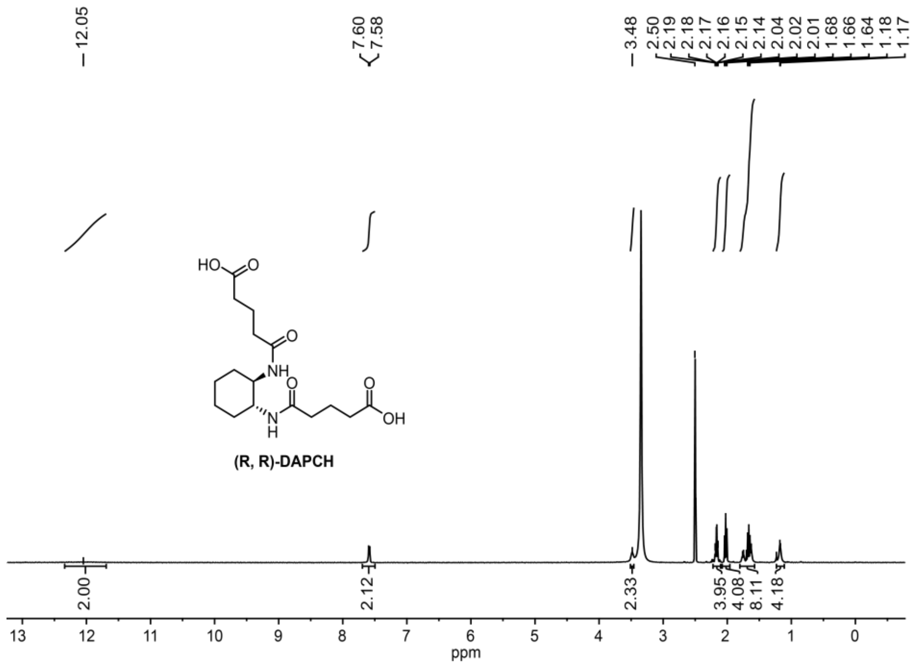

Supplementary Figure 7. ^{1}H NMR spectrum of **(R, R)-DAPCH**.

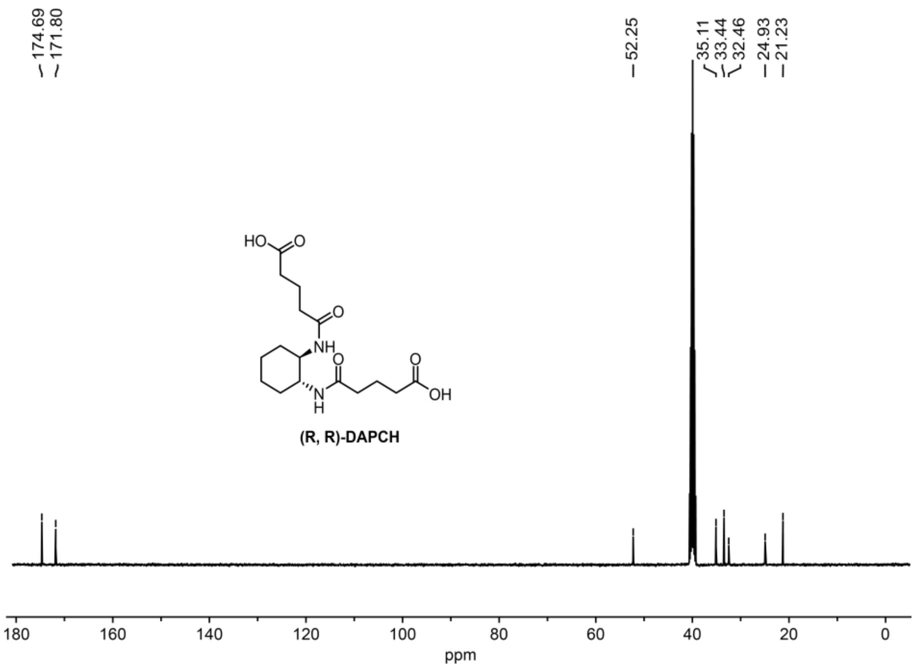

Supplementary Figure 8. ^{13}C NMR spectrum of **(R, R)-DAPCH**.

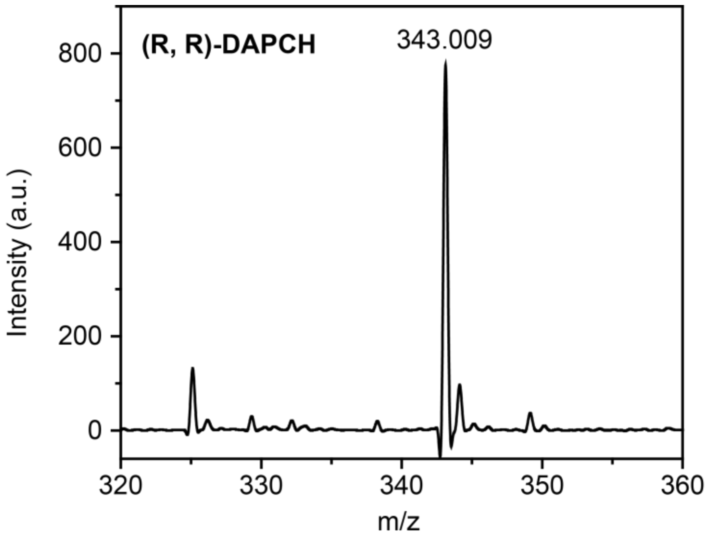

Supplementary Figure 9. MALDI-TOF spectrum of **(R, R)-DAPCH**.

Synthesis of *trans*-(1*S*, 2*S*)-cyclohexanedi (3-aminocarbonylbutanoic) acid ((S, S)-DAPCH)

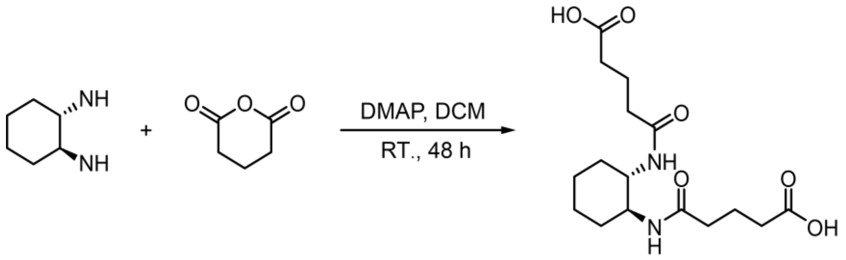

(S, S)-DAPCH was prepared under the identical synthetic conditions described in the preparation of **(R, R)-DAACH** using *trans*-(1*S*, 2*S*)-diamidocyclohexane (1.00 g, 8.76 mmol), 4-dimethylaminopyridine (20 mg, 0.18 mmol) and glutaric anhydride (2.09 g, 18.4 mmol). Yield: 2.65 g of white powder (88%). m.p.: 190°C. ^{1}H NMR (400 MHz, DMSO-d_6, ppm): δ 12.00 (s, 2H), 7.60-7.58 (d, J= 7.9 Hz, 2H), 3.48 (s, 2H), 2.19-2.14 (m, 4H), 2.04-2.01 (t, J = 7.4 Hz, 4H), 1.76-1.63 (m, 8H), 1.23-1.17 (t, J = 12.9 Hz, 4H); ^{13}C NMR (100 MHz, DMSO-d_6, ppm): δ 174.69, 171.80, 52.26, 35.11, 33.44, 32.46, 24.93, 21.23. MALDI-TOF: m/z calcd for $C_{16}H_{27}N_2O_6$: 343.186 $[M+H]^+$; found: 343.130.

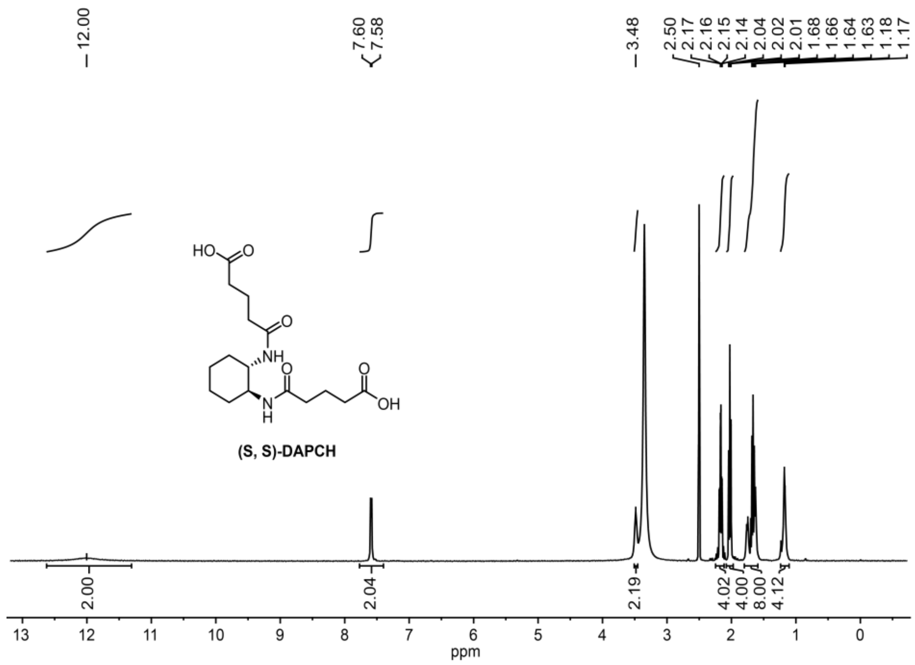

Supplementary Figure 10. ^{1}H NMR spectrum of **(S, S)-DAPCH**.

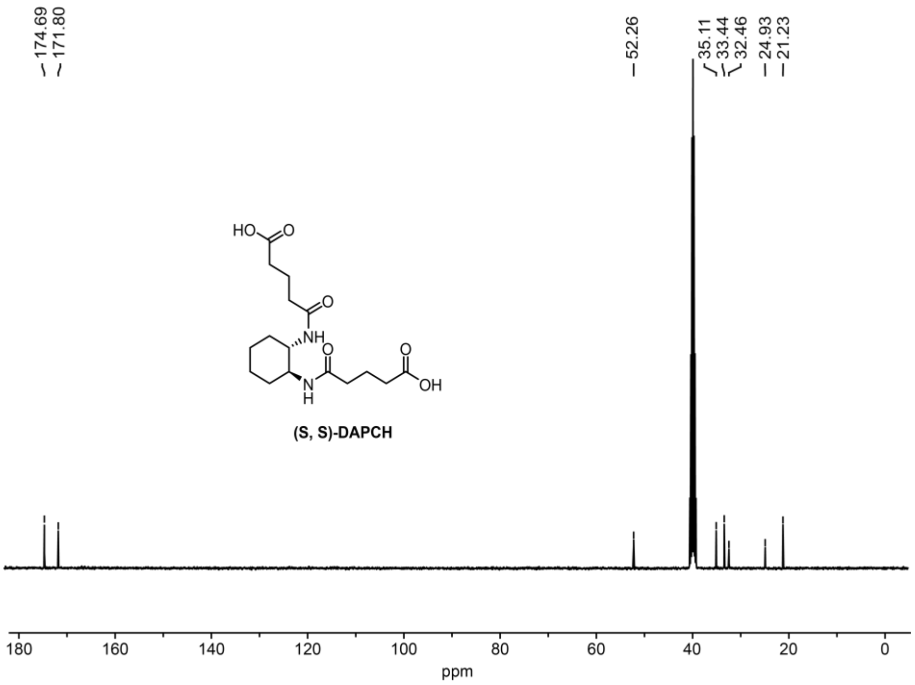

Supplementary Figure 11. ^{13}C NMR spectrum of **(S, S)-DAPCH**.

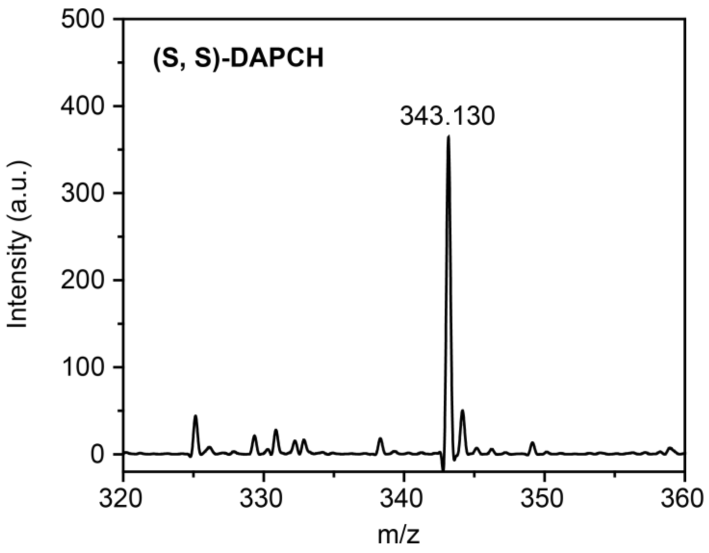

Supplementary Figure 12. MALDI-TOF spectrum of **(S, S)-DAPCH**.

2. Thermal properties

Thermogravimetric analyses (TGA) were conducted on a DTG-60 Shimadzu thermal analyst system under a heating rate of 10ºC/min and a nitrogen flow rate of 50 cm^3/min. The differential scanning calorimetry (DSC) analyses were performed on a Shimadzu DSC-60A instrument under a heating rate of 10ºC/min and a nitrogen flow rate of 20 cm^3/min.

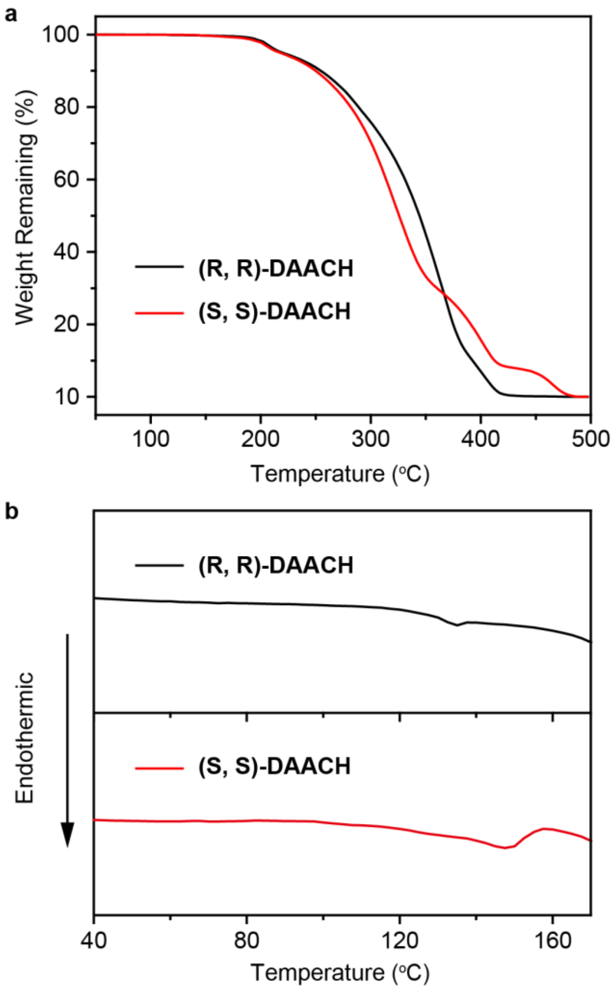

Supplementary Figure 13. (a) TGA and (b) DSC curves of **(R, R)-DAACH** and **(S, S)-DAACH**.

3. Photophysical investigations

Fluorescence spectra were recorded on Edinburgh FLS980. The absolute photoluminescence quantum yield (PLQY) was obtained using an Edinburgh FLS980 fluorescence spectrophotometer equipped with an integrating sphere. Phosphorescence spectra were obtained using an Edinburgh FLS980 fluorescence spectrophotometer with a 10 ms delay time after excitation using a microsecond flash lamp. The microsecond flash lamp produces short, typically a few μs, and high irradiance optical pulses for phosphorescence decay measurements in the range from microseconds to seconds. The kinetic measurements, afterglow spectra and ultralong lifetimes were also measured using an Edinburgh FLS980 fluorescence spectrophotometer. Excitation-phosphorescence mapping was measured using Hitachi F-4700 with a 25 ms delay time under ambient condition. The intrinsic circularly polarized luminescence (CPL) spectra were investigated using a JASCO CPL-300 spectrometer.

To individually achieve the fluorescence and phosphorescence signal of CPL emission, the CPL spectra were split into fluorescence and phosphorescence parts through using the short-pass and long-pass filters owing to the large stokes-shift of the fluorescence and phosphorescence in organic afterglow materials. Experimentally, when we measured the fluorescence component of CPL emission, the short-pass filter was used to eliminate the influence of the phosphorescence emission on the fluorescence signal of CPL emission; and, the long-pass filter was employed to suppress the influence of the fluorescence emission on the phosphorescence signal of CPL emission. The circular dichroism (CD) spectra were measured on a JASCO J-810 circular dichroism spectrometer with 'Low' sensitivity. The scan speed was set as 200 nm/min with 1 nm resolution and a respond time of 1.0 s. Experimentally, the dissymmetry factor (g_{lum}) can be calculated from following equation (1): $g_{\text{lum}}=2(I_L-I_R)/(I_L+I_R)$. Theoretically, g_{lum} is defined as:

$$g_{\text{lum}}=\frac{4|m|\cos\theta}{|\mu|} \tag{2}$$

where $|m|$ and $|\mu|$ are the magnitudes of magnetic and electric transition dipole moments vectors, respectively, and the θ is the angle between these two dipole moments. Therefore, large $|\mu|$ means small g_{lum}, while large $|m|$ will result in a high g_{lum} value[2,3].

The lifetimes (τ) of the luminescence were obtained by fitting the decay curve with a multi-exponential decay function of

$$I(\mathrm{t}) = \sum_i A_i e^{-\frac{t}{\tau_i}} \tag{3}$$

where A_i and τ_i represent the amplitudes and lifetimes of the individual components for multi-exponential decay profiles, respectively[4].

The solution of **(R, R)-DAACH** with varied concentrations can be facilely prepared by dissolution of **(R, R)-DAACH** powder in ethanol (99.5% purity) followed by the sonication for 10 min under ambient conditions.

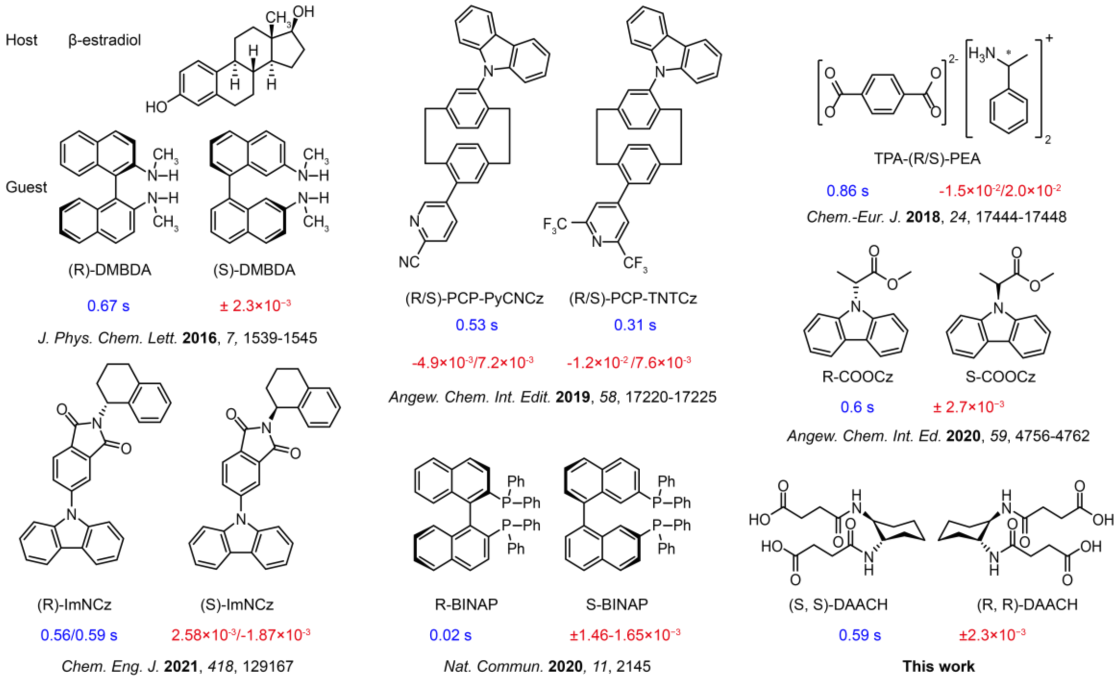

Supplementary Figure 14. Reported chiral organic afterglow materials with corresponding lifetimes (blue) and g_{lum} (red) under ambient conditions[5-10].

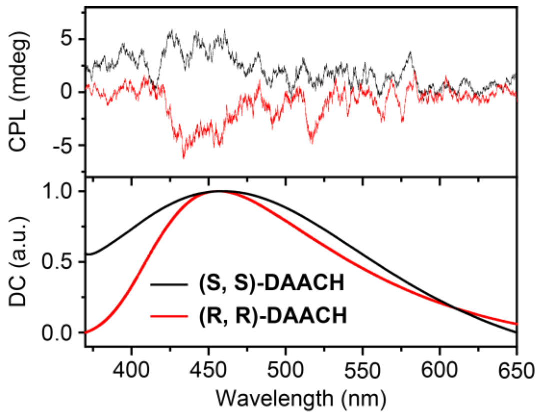

Supplementary Figure 15. CPL properties of the steady-state photoluminescence (SSPL) of **(R, R)-DAACH** and **(S, S)-DAACH** (50 wt%)-dispersed in potassium bromide (KBr) slices when excited by 300 nm under ambient conditions.

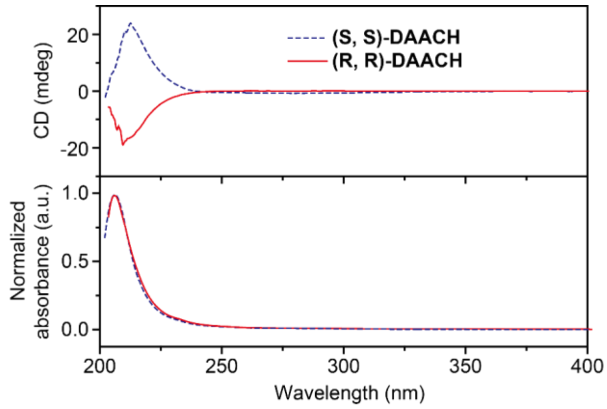

Supplementary Figure 16. Circular dichroism (top) and UV-absorption (bottom) spectra of **(S, S)-DAACH** and **(R, R)-DAACH** in ethanol solutions (10^{-4} M) under ambient conditions.

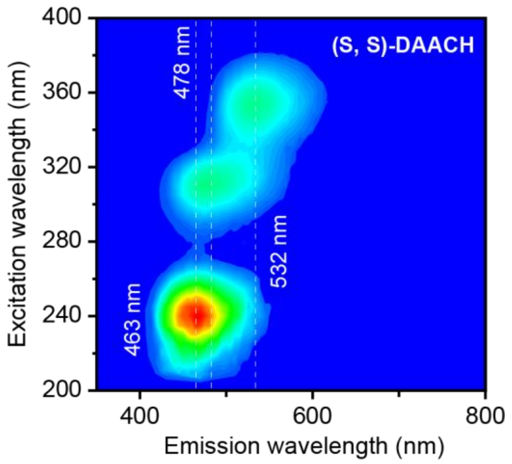

Supplementary Figure 17. Excitation-phosphorescence mapping of **(S, S)-DAACH** powder with a delay time of 25 ms under ambient conditions.

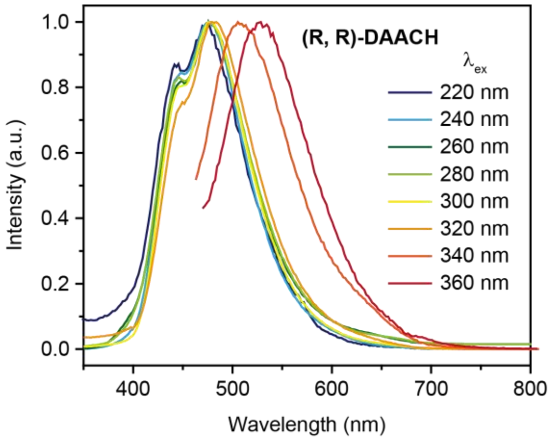

Supplementary Figure 18. Afterglow spectra of **(R, R)-DAACH** powder upon excitation at different wavelength under ambient conditions.

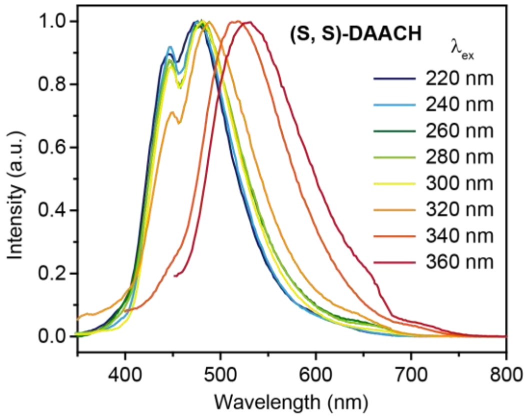

Supplementary Figure 19. Afterglow spectra of **(S, S)-DAACH** powder upon excitation at different wavelength under ambient conditions.

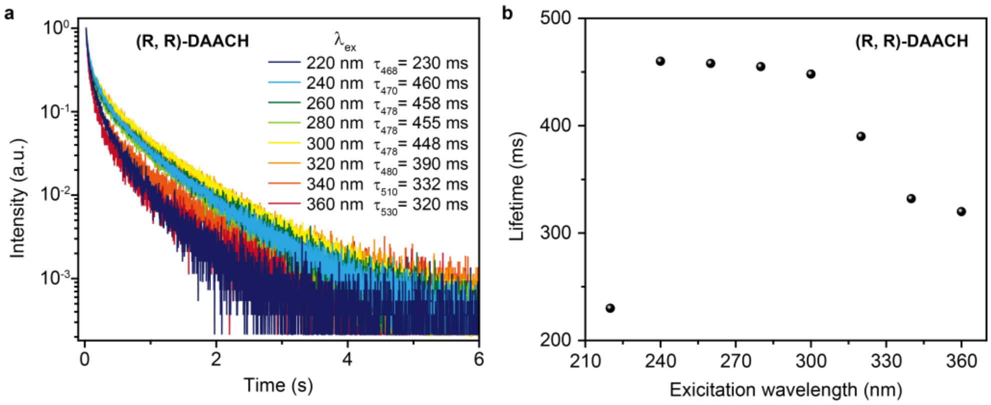

Supplementary Figure 20. (a) Afterglow decay profiles and (b) corresponding lifetimes of the main emission bands of **(R, R)-DAACH** powder upon excitation at different wavelength under ambient conditions.

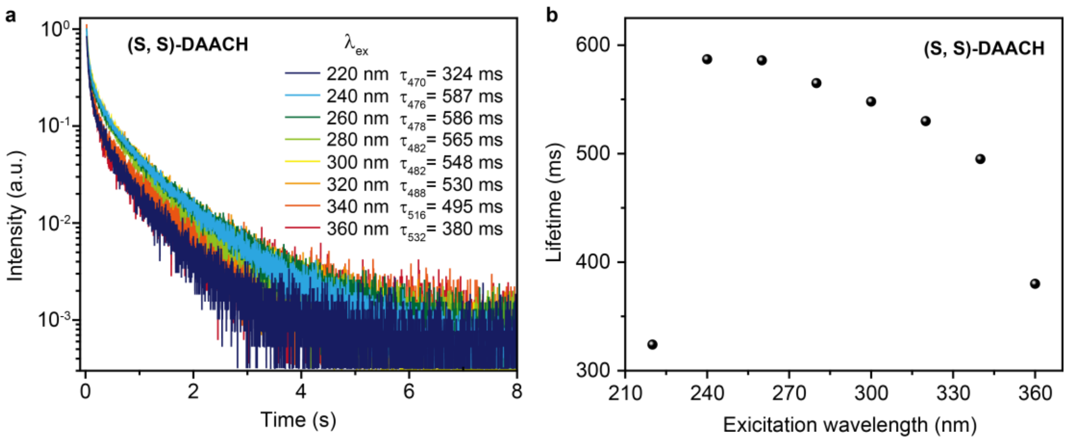

Supplementary Figure 21. (a) Afterglow decay profiles and (b) corresponding lifetimes of the main emission bands of **(S, S)-DAACH** powder upon excitation at different wavelength under ambient conditions.

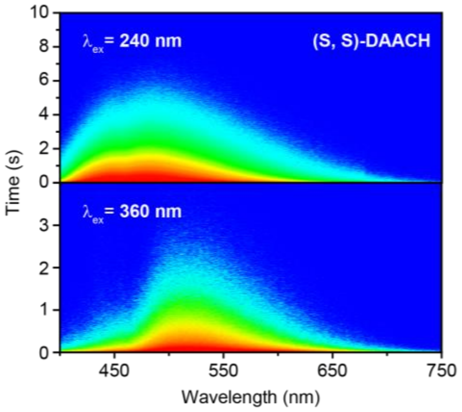

Supplementary Figure 22. Transient photoluminescence decay images of **(S, S)-DAACH** upon excitation at 240 and 360 nm, respectively.

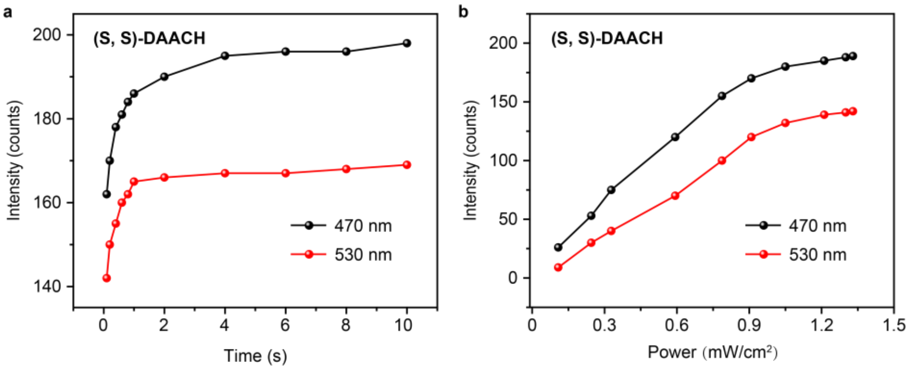

Supplementary Figure 23. Changes in CPOA intensities of **(S, S)-DAACH** powder at 470 and 530 nm as a function of (a) irradiation time and (b) intensity upon excitation by 240 (black line) and 360 nm (red line), respectively.

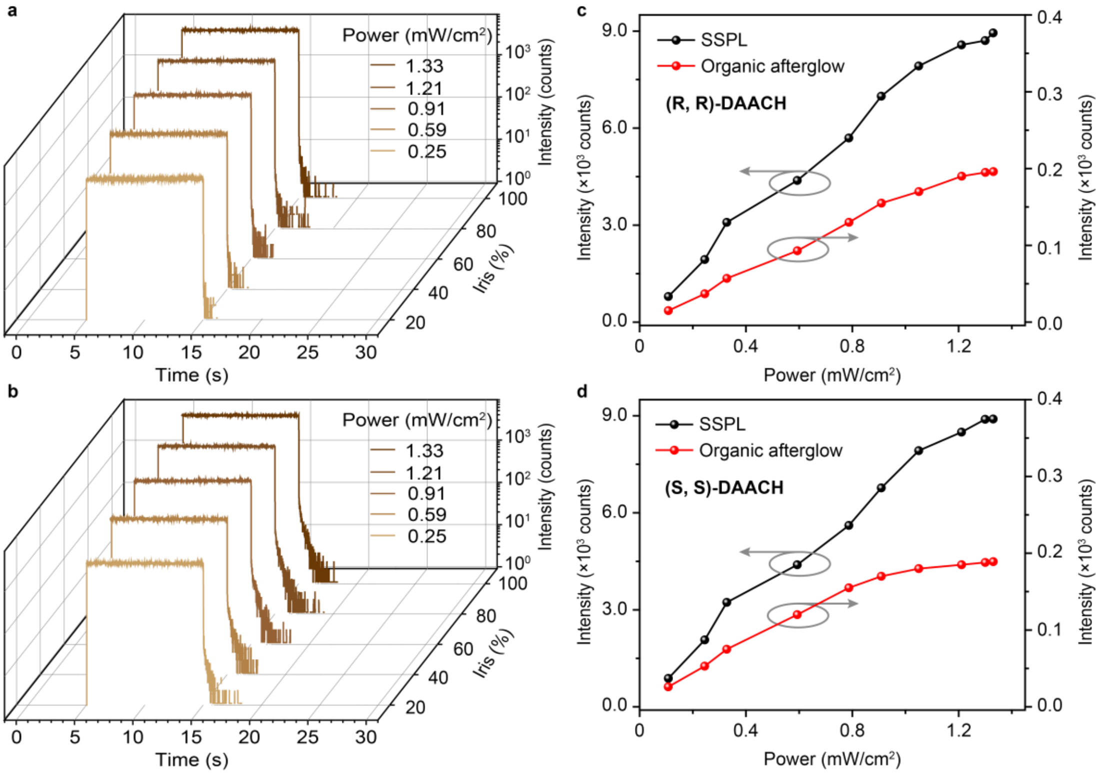

Supplementary Figure 24. (a-b) Photoluminescence intensity profiles of 470 nm emission of (a) **(R, R)-DAACH** and (b) **(S, S)-DAACH** powders at different excitation power density of 240 nm irradiation. (c-d) SSPL (black) and organic afterglow (red) intensities as a function of the excitation power under ambient conditions.

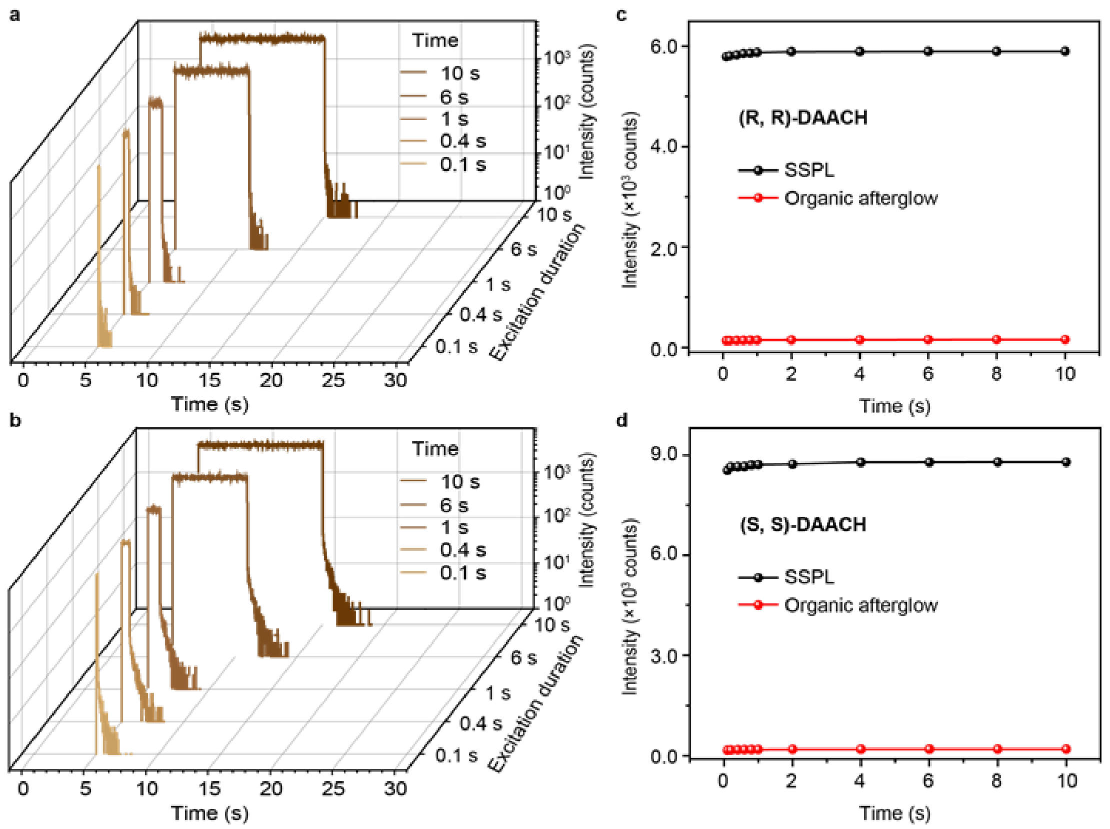

Supplementary Figure 25. (a-b) Photoluminescence intensity profiles of 470 nm emission of (a) **(R, R)-DAACH** and (b) **(S, S)-DAACH** powders as a function of time upon excitation with different irradiating time (λ_{ex} = 240 nm) under ambient conditions. (c-d) Corresponding SSPL (black) and organic afterglow (red) intensities with different irradiating time (0.1 ~ 10 s).

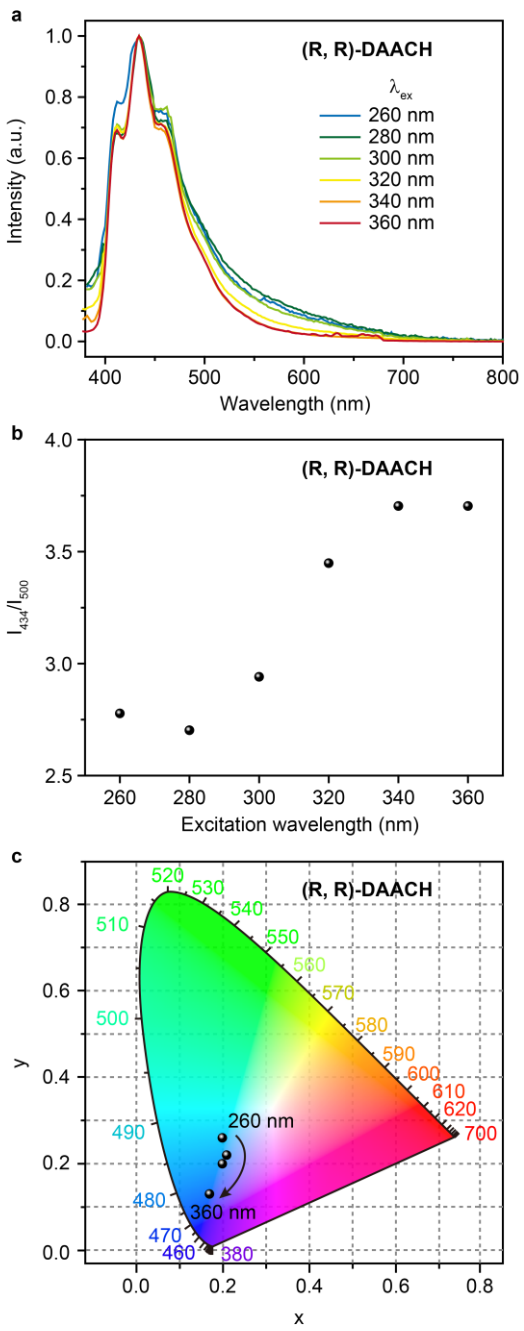

Supplementary Figure 26. (a) SSPL spectra, (b) emission intensity ratios between 434 and 500 nm and (c) CIE coordinates of **(R, R)-DAACH** powder upon excitation at different wavelength under ambient conditions.

Supplementary Table 1. PLQYs of **(R, R)-DAACH** and **(S, S)-DAACH** upon excitation at different wavelength under ambient conditions.

Parameters	**(R, R)-DAACH**	**(S, S)-DAACH**
280 nm	2.54%	3.38%
320 nm	2.74%	6.31%
360 nm	4.56%	10.16%

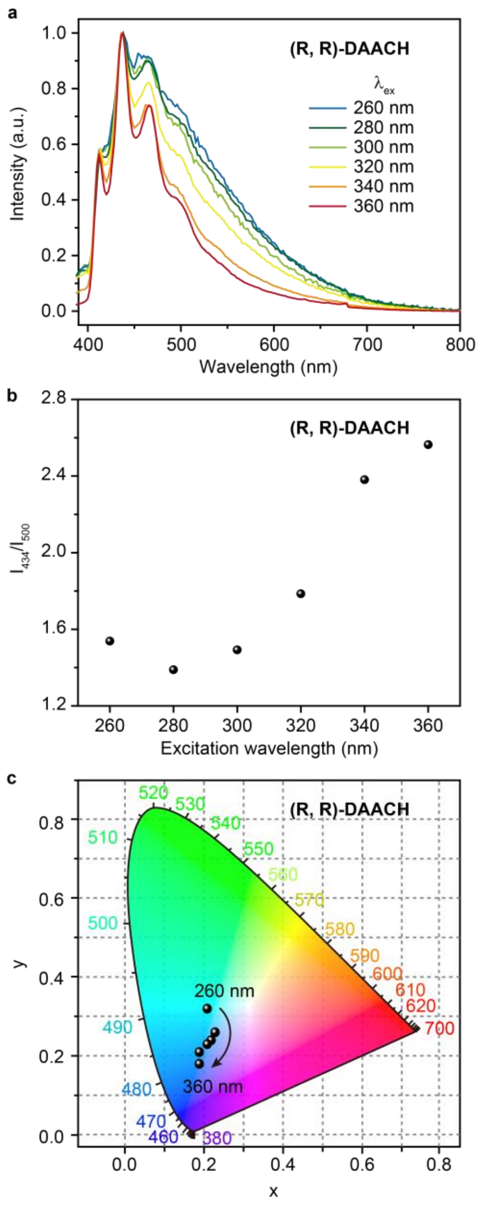

Supplementary Figure 27. (a) SSPL spectra, (b) emission intensity ratios between 434 and 500 nm and (c) CIE coordinates of **(R, R)-DAACH** powder upon excitation at different wavelength at 77 K.

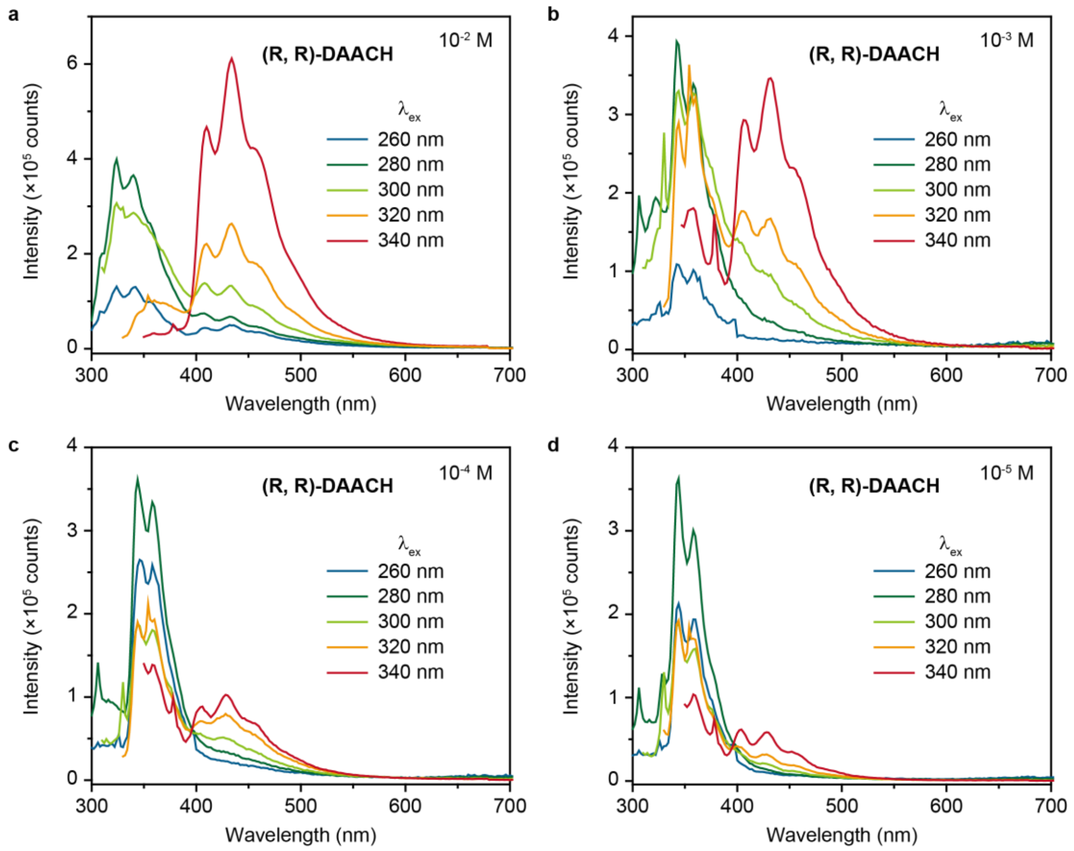

Supplementary Figure 28. SSPL spectra of **(R, R)-DAACH** in ethanol solutions with varied concentrations of (a) 10^{-2}, (b) 10^{-3}, (c) 10^{-4} and (d) 10^{-5} M upon excitation at different wavelength under ambient conditions.

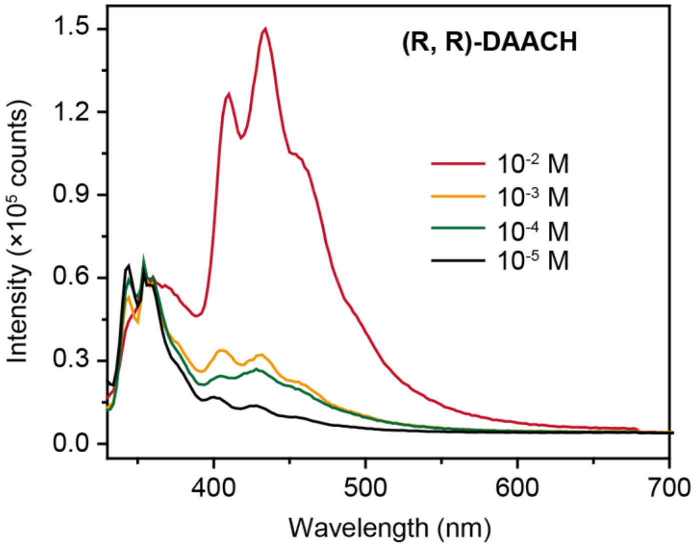

Supplementary Figure 29. SSPL spectra of **(R, R)-DAACH** in ethanol solutions with varied concentrations excited by 320 nm under ambient conditions.

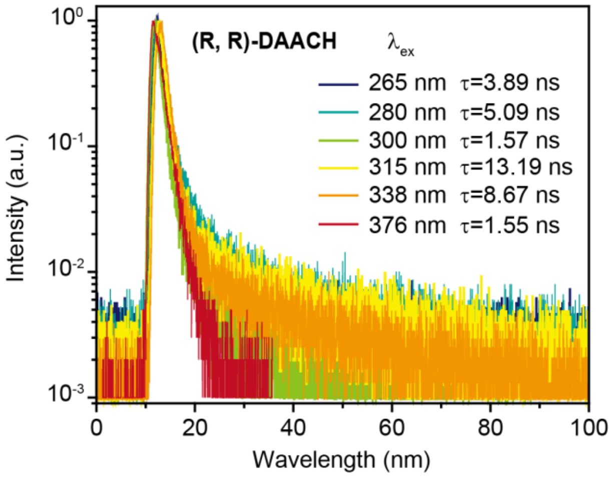

Supplementary Figure 30. Fluorescence decay profiles of emission band at 434 nm of **(R, R)-DAACH** in ethanol solution (10^{-2} M) upon excitation at different wavelength under ambient conditions.

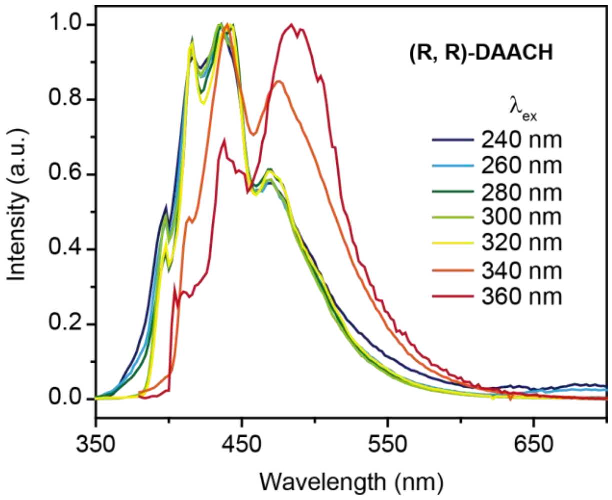

Supplementary Figure 31. Phosphorescence spectra of **(R, R)-DAACH** in ethanol solution (10^{-2} M) upon excitation at different wavelength recorded at 77 K.

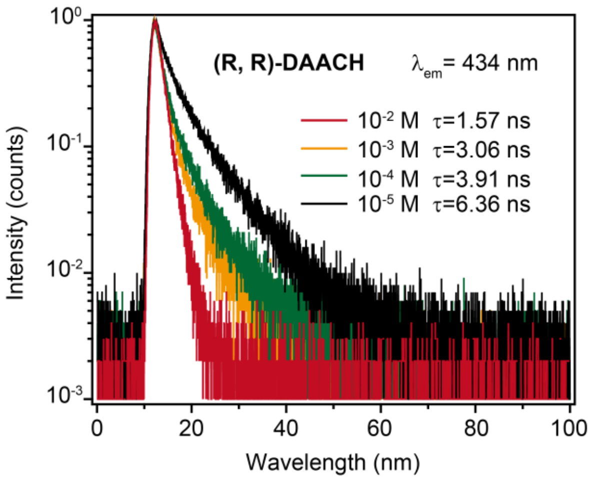

Supplementary Figure 32. Time-resolved decay profiles of emission band (434 nm) at varied concentrations in ethanol solution of **(R, R)-DAACH** excited by 300 nm under ambient conditions.

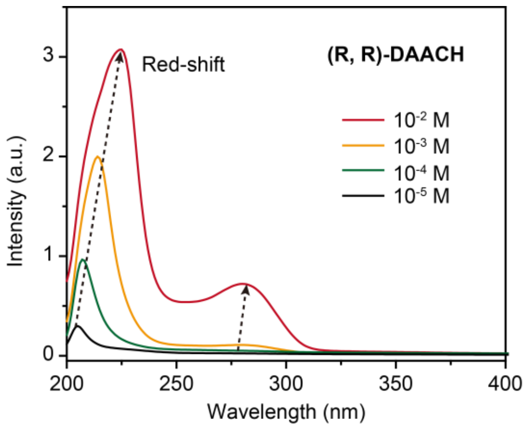

Supplementary Figure 33. Absorption spectra of **(R, R)-DAACH** in ethanol solutions with varied concentrations of 10^{-2}, 10^{-3}, 10^{-4} and 10^{-5} M under ambient conditions.

4. Single crystals analysis

Colorless single crystals of **(R, R)-DAACH** was obtained by slow evaporation of a mixed deionized water and ethanol solution under ambient condition. All the data of single crystal structures were collected on a Bruker SMART APEX (II)-CCD at 100 K and crystal structures were analyzed by Mercury 4.0 software. Single crystal data were summarized in **Supplementary Table 2**. The analyses of independent gradient model (IGM) for intermolecular weak interactions were carried out by Multiwfn 3.6 and were volume rendered by VMD 1.9.3 based on the crystal structure of **(R, R)-DAACH**.

Supplementary Table 2. Crystallographic data of **(R, R)-DAACH** at 100 K.

Compound	**(R, R)-DAACH**
Formula	$C_{14}H_{22}N_2O_6$
Formula weight (g mol^{-1})	314.33
Crystal color	colorless
Wavelength (Å)	0.71073
Crystal system	monoclinic
Space group	P21
a, (Å)	11.523(8)
b, (Å)	4.895(4)
c, (Å)	15.044(10)
α, (deg)	90
β, (deg)	109.667(16)
γ, (deg)	90
volume, (Å^3)	799.1(10)
Z	2
Density, (g cm^{-3})	1.306
μ, (mm^{-1})	0.102
F(000)	336
h_{max}, k_{max}, l_{max}	13, 5, 17
$Theta_{max}$	27.876
CCDC number	2083718

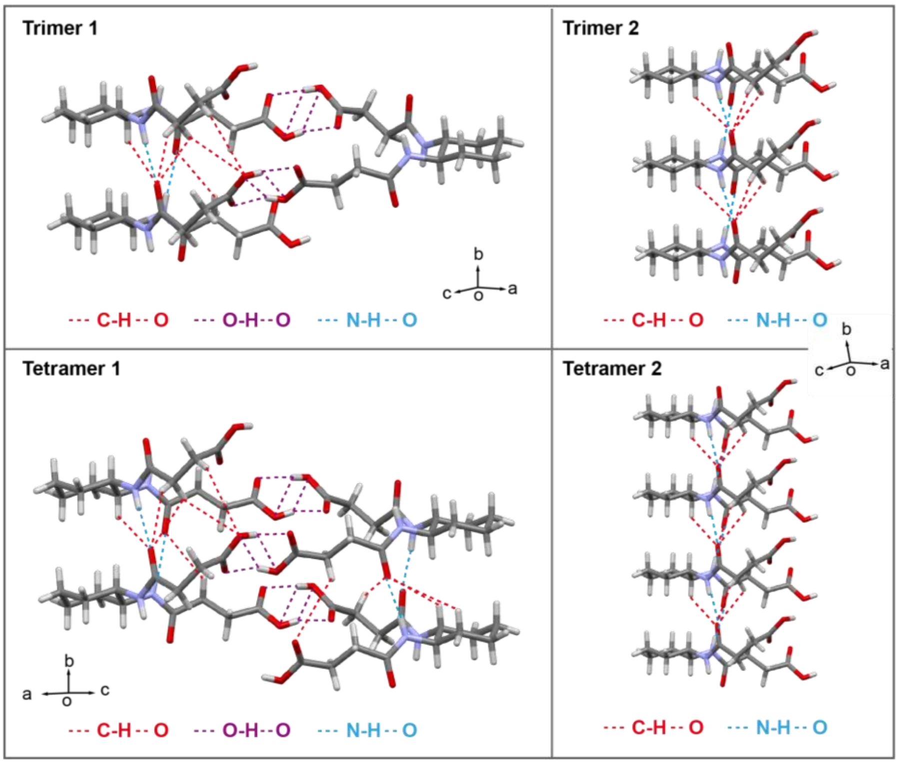

Supplementary Figure 34. Molecular packing arrangements showing detailed intermolecular interactions of selected trimmers and tetramers extracted from **(R, R)-DAACH** single crystal.

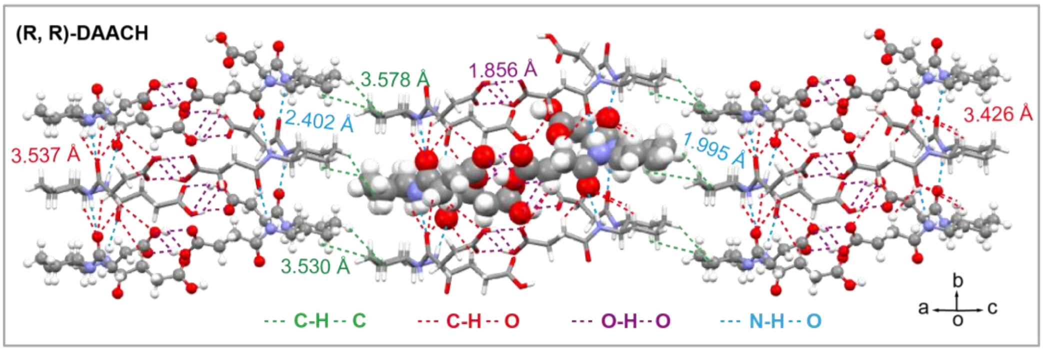

Supplementary Figure 35. Molecular packing arrangements showing detailed intermolecular interactions in **(R, R)-DAACH** single crystal.

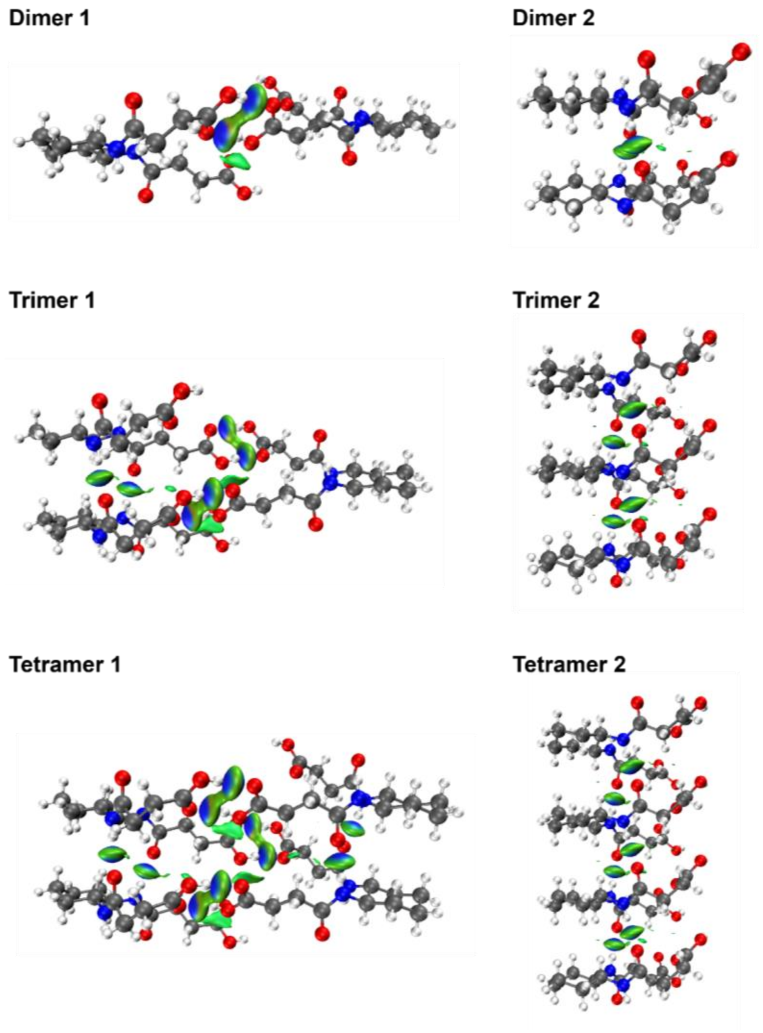

Supplementary Figure 36. Calculated independent gradient model (IGM) (green isosurface) of selected dimers, trimmers and tetramer extracted from **(R, R)-DAACH** single crystal (the isovalue is 0.01).

5. Theoretical calculations

Density functional theory (DFT) and time-dependent DFT (TD-DFT) simulations were performed with Gaussian 09 package[11,12]. All the computational models were built from the single-crystal structures without further geometry optimization. The excitation energy of the *n*-th singlet state (S_n) and the *n*-th triplet state (T_n) states were calculated at TD-DFT method of B3LYP/6-31G(d, p) level based on the monomer and selected aggregates extracted from the single-crystal. Frontier molecular orbital distributions were calculated based on the monomer and selected aggregates extracted from the single-crystal at M06-2X/cc-pVTZ. Electron density differences (EDD) upon the $S_0 \rightarrow S_1$ and $S_0 \rightarrow T_1$ transitions based on the single crystal structures were carried out using Multiwfn by subtracting the electron density of grounded-state (S_0) from that of interested singlet or triplet excited states.

Supplementary Table 3. TD-DFT predicted singlet and triplet excited state energies and transition configurations of monomer of **(R, R)-DAACH**.

Excited state	*n*-th	Energy (eV)	Transition configuration (%)
S_n	1	5.6490	H→L+9 (38.2), H→L+10 (10.0), H→L+6 (9.0), H→L+5 (8.6), H→L+7 (7.5), H→L+2 (6.5), H→L+4 (6.3)
T_n	1	5.1749	H→L+9 (37.8), H→L+10 (9.7), H→L+5 (7.4), H→L+6 (7.1), H→L+7 (6.8), H-4→L+3 (5.2)
	2	5.1958	H-4→L+3 (43.2), H-4→L+2 (37.8), H→L+9 (5.9)
	3	5.2539	H-1→L+9 (27.9), H-3→L+9 (10.7), H-1→L+10 (7.6), H-1→L+7 (6.4), H-1→L+6 (5.6)
	4	5.2659	H-3→L+7 (22.3), H-1→L+7 (15.0), H-2→L+7 (6.0), H-3→L+6 (5.8)
	5	5.2910	H-2→L+7 (34.5), H-3→L+7 (9.7), H-2→L+6 (9.2), H-2→L+5 (6.3)
	6	5.3372	H-5→L (59.7), H-5→L+1 (33.2)
	7	5.8287	H-6→L+3 (48.8), H-6→L+2 (42.1)
	8	6.0010	H-9→L (25.5), H-8→L (23.6), H-9→L+1 (15.1), H-8→L+1 (13.8)
	9	6.0422	H-2→L (43.7), H-1→L (22.2), H-2→L+1 (10.6), H→L (9.4)
	10	6.0884	H→L (64.9), H→L+1 (15.3), H-2→L (5.5)

Supplementary Table 4. TD-DFT predicted singlet and triplet excited state energies and transition configurations of selected dimer of **(R, R)-DAACH**.

Excited state	*n*-th	Energy (eV)	Transition configuration (%)
S_n	1	5.6038	H→L (73.3), H→L+20 (5.6), H→L+16 (5.3)
T_n	1	5.1899	H-6→L+9 (29.5), H→L+20 (8.6), H-6→L+7 (7.0), H→L+16 (5.9), H-8→L+9 (5.7), H→L+22 (5.1)
	2	5.1973	H-7→L+6 (40.7), H-7→L+7 (22.3), H-7→L+9 (5.1)
	3	5.2071	H-6→L+9 (19.8), H→L+20 (13.8), H→L+16 (8.9), H→L+22 (8.3), H-1→L+20 (5.6)
	4	5.2299	H-1→L+20 (21.3), H-1→L+22 (13.5), H-1→L+16 (13.2), H→L+20 (9.0), H→L+16 (6.0), H→L+22 (5.2)
	5	5.2343	H-3→L+19 (15.0), H-2→L+19 (9.7)
	6	5.2510	H-8→L+5 (20.3), H-8→L+8 (9.3), H-8→L+7 (6.0), H-6→L+5 (5.1)
	7	5.2730	H-11→L+1 (45.7), H-11→L (45.6)
	8	5.2833	H-5→L+12 (21.2), H-5→L+13 (16.4), H-5→L+6 (16.2), H-5→L+11 (7.2)
	9	5.3008	H-2→L+19 (18.1), H-3→L+19 (14.4), H-2→L+1 (5.4), H-2→L+20 (5.1)
	10	5.3362	H-10→L+4 (71.1), H-10→L+2 (10.1)
	11	5.3543	H-4→L+12 (24.2), H-4→L+6 (20.9), H-4→L+13 (18.2), H-4→L+11 (8.3)
	12	5.4414	H-9→L+5 (34.3), H-9→L+8 (16.1), H-9→L+7 (10.0), H-9→L (7.4), H-9→L+3 (6.9)
	13	5.6237	H→L (90.8), H→L+1 (5.2)
	14	5.7129	H→L+1 (82.7), H→L+2 (6.2)
	15	5.7869	H-1→L (91.1)
	16	5.8154	H-12→L+6 (28.5), H-13→L+6 (18.9), H-12→L+7 (15.4), H-13→L+7 (10.0)
	17	5.8284	H-13→L+9 (33.8), H-12→L+9 (27.2), H-13→L+7 (8.0), H-12→L+7 (6.1)
	18	5.8418	H-2→L+4 (26.1), H-2→L+1 (21.0), H-2→L (17.7), H-2→L+2 (15.0)
	19	5.8606	H-1→L+1 (81.1), H-1→L+2 (10.0)
	20	5.8974	H-2→L (71.5), H-2→L+1 (14.9)
	21	5.9267	H-3→L (63.6), H-19→L+1 (10.9), H-19→L (10.7)

Supplementary Table 5. TD-DFT predicted singlet and triplet excited state energies and transition configurations of selected trimer 1 of **(R, R)-DAACH**.

Excited state	*n*-th	Energy (eV)	Transition configuration (%)
S_n	1	5.5870	H→L (92.2)
T_n	1	5.1819	H-4→L+18 (45.5), H-4→L+21 (9.9), H-4→L+19 (6.2)
	2	5.1941	H→L+29 (11.5), H→L+27 (10.9), H→L+26 (10.1), H→L+24 (5.0)
	3	5.2189	H-10→L+13 (20.5), H-10→L+16 (14.7), H-10→L+14 (13.3), H-10→L+15 (10.4)
	4	5.2279	H-1→L+29 (11.5), H-1→L+27 (10.7), H-1→L+26 (9.8), H→L+29 (5.2), H→L+27 (5.0)
	5	5.2397	H-8→L+18 (18.1), H-6→L+18 (14.3), H-5→L+18 (13.5)
	6	5.2436	H-11→L+9 (10.5), H-11→L+10 (9.9), H-3→L+26 (7.2), H-11→L+12 (7.1), H-11→L+1 (5.1)
	7	5.2571	H-3→L+26 (9.2), H-11→L+9 (7.9), H-11→L+10 (7.8), H-2→L+26 (5.2)
	8	5.2641	H-5→L+16 (17.8), H-5→L+21 (13.9), H-5→L+17 (7.1)
	9	5.2710	H-9→L+17 (21.4), H-9→L+14 (15.0), H-9→L+19 (8.9), H-9→L+7 (5.2), H-9→L+11 (5.0)
	10	5.2971	H-6→L+16 (14.3), H-6→L+21 (11.1), H-8→L+16 (9.0), H-8→L+21 (7.1), H-6→L+17 (5.7)
	11	5.3002	H-2→L+26 (16.6), H-3→L+26 (10.1), H-2→L+29 (7.8), H-2→L+28 (5.9)
	12	5.3390	H-13→L+8 (27.6), H-13→L+7 (21.6), H-13→L+6 (14.2), H-13→L+11 (12.0), H-13→L+9 (5.9)
	13	5.3593	H-7→L+17 (24.0), H-7→L+14 (17.0), H-7→L+19 (9.5), H-7→L+7 (6.6), H-7→L+11 (6.6), H-7→L+6 (5.4)
	14	5.4337	H-12→L+9 (21.8), H-12→L+10 (20.2), H-12→L+12 (13.8), H-12→L+1 (12.2)
	15	5.4406	H-14→L+2 (40.8), H-14→L+7 (9.0), H-14→L+8 (8.5), H-14→L+10 (6.6)
	16	5.4488	H-15→L (34.4), H-15→L+5 (26.5), H-17→L+5 (8.2), H-17→L (6.1)
	17	5.5170	H-17→L (55.0), H-15→L+5 (21.6)
	18	5.5718	H-16→L+2 (42.6), H-14→L+2 (9.7), H-14→L+10 (5.8)
	19	5.5939	H→L (98.0)
	20	5.7361	H→L+1 (87.8)
	21	5.7605	H-1→L (93.7)

	22	5.8034	H-2→L (91.8)
	23	5.8267	H-18→L+13 (23.3), H-18→L+16 (17.3), H-18→L+14 (15.4), H-18→L+15 (12.1), H-18→L+17 (5.7)
	24	5.8634	H-3→L (95.6)
	25	5.8826	H-1→L+1 (83.9)
	26	5.9328	H→L+2 (92.4)
	27	5.9378	H-19→L+2 (16.8), H-19→L+5 (11.4), H-24→L+5 (6.1)
	28	5.9471	H-19→L+2 (9.8), H-24→L+5 (8.1), H-19→L+7 (7.4), H-19→L+8 (5.4)
	29	5.9491	H-2→L+1 (45.5), H-2→L+6 (11.4), H-2→L+7 (6.0), H-2→L+8 (5.9)
	30	5.9809	H-3→L+1 (40.7), H-2→L+8 (9.8), H-2→L+1 (7.7), H-2→L+7 (7.5), H-2→L+3 (5.1)

Supplementary Table 6. TD-DFT predicted singlet and triplet excited state energies and transition configurations of selected trimer 2 of **(R, R)-DAACH**.

Excited state	*n*-th	Energy (eV)	Transition configuration (%)
S_n	1	5.5369	H→L (60.5), H→L+1 (32.0)
T_n	1	5.1874	H-11→L+13 (32.3), H-11→L+12 (18.2), H-11→L+14 (11.6)
	2	5.1990	H-12→L+11 (51.1), H-12→L+10 (12.7), H-12→L+12 (5.2)
	3	5.2023	H-10→L+14 (28.5), H-10→L+13 (17.7), H-10→L+17 (12.0), H-10→L+16 (10.9)
	4	5.2080	H→L+31 (11.2), H→L+26 (9.4), H-11→L+13 (8.9), H-1→L+31 (6.0), H-11→L+12 (5.1)
	5	5.2122	H-6→L+19 (10.6), H-6→L+21 (9.4), H-6→L+18 (8.5), H-6→L+20 (7.7), H-6→L+6 (5.1)
	6	5.2325	H-1→L+31 (13.5), H-1→L+26 (9.9), H→L+31 (7.2), H→L+26 (6.1), H-1→L+27 (5.4)
	7	5.2405	H-13→L+9 (27.1)
	8	5.2435	H-3→L+29 9.9), (H-13→L+9 9.0), (H-2→L+29 (8.5)
	9	5.2507	H-5→L+25 (9.5), H-5→L+22 (7.9), H-5→L+24 (6.2)
	10	5.2608	H-17→L+1 (77.6), H-17→L (9.1)
	11	5.2727	H-16→L+4 (38.4), H-16→L+2 (18.6), H-16→L+3 (18.5), H-16→L+5 (6.7), H-16→L (5.9)
	12	5.2847	H-8→L+18 (8.3), H-9→L+18 (6.6)
	13	5.3086	H-2→L+29 (14.3), H-3→L+29 (12.6), H-2→L+24 (7.4), H-3→L+24 (6.0)
	14	5.3351	H-15→L+5 (37.7), H-15→L+8 (36.3), H-15→L+3 (6.7)
	15	5.3584	H-7→L+18 (17.9), H-7→L+20 (9.8), H-7→L+11 (8.6), H-7→L+17 (6.8), H-7→L+12 (5.5)
	16	5.3993	H-4→L+25 (11.2), H-4→L+22 (9.0), H-4→L+24 (8.0), H-5→L+25 (6.6), H-5→L+22 (5.4)
	17	5.4485	H-14→L+9 (37.1), H-14→L (6.8), H-14→L+10 (5.5)
	18	5.4762	H-9→L+19 (7.6), H-9→L+18 (6.5), H-9→L+21 (6.4), H-8→L+19 (6.1), H-9→L+20 (5.3), H-8→L+18 (5.2), H-8→L+21 (5.1)
	19	5.5468	H→L (56.8), H→L+1 (38.4)
	20	5.6150	H→L+1 (55.0), H→L (26.6), H→L+2 (6.8), H→L+3 (5.3)
	21	5.7040	H-1→L (62.9), H-1→L+1 (30.3)
	22	5.7568	H-1→L+1 (62.3), H-1→L (18.7), H-1→L+2 (9.4)
	23	5.8033	H→L+2 (67.0), H→L (11.1)
	24	5.8163	H-19→L+11 (32.8), H-20→L+11 (16.8), H-19→L+10 (8.2),

			H-18→L+11 (6.9)
	25	5.8177	H-18→L+14 (28.5), (H-18→L+13 (17.9), H-18→L+17 (13.2), H-18→L+16 (11.5), H-20→L+14 (7.0)
	26	5.8185	H-2→L (28.9), H-2→L+3 (17.0), H-2→L+5 (15.9), H-2→L+1 (9.0), H-2→L+8 (7.9)
	27	5.8243	H-20→L+13 (20.4), H-19→L+13 (17.5), H-20→L+12 (11.1), H-19→L+12 (9.8), H-20→L+14 (6.8), H-19→L+14 (6.4)
	28	5.8770	H-3→L (33.8), H-2→L (15.7), H-3→L+1 (7.6)
	29	5.8786	H→L+4 (28.6), H→L+3 (23.8), H→L+2 (14.5), H-3→L (5.1)
	30	5.9025	H-3→L (18.1), H-2→L (17.0), H-2→L+8 (9.7), H-2→L+5 (8.0), H-4→L (7.5), H-2→L+4 (6.9)
	31	5.9087	H-4→L+1 (47.7), H-4→L (17.6), H-30→L+1 (8.0), H-3→L+1 (6.0)

Supplementary Table 7. TD-DFT predicted singlet and triplet excited state energies and transition configurations of selected tetramer 1 of **(R, R)-DAACH**.

Excited state	*n*-th	Energy (eV)	Transition configuration (%)
S_n	1	5.5212	H→L (47.1), H→L+1 (46.4)
T_n	1	5.1862	H-16→L+17 (49.1), H-16→L+16 (10.1), H-16→L+15 (7.2)
	2	5.1971	H-13→L+20 (23.5), H-13→L+18 (12.3), H-13→L+19 (11.1), H-13→L+25 (6.9), H-13→L+21 (6.3)
	3	5.2000	H-17→L+16 (35.0), H-17→L+15 (27.2)
	4	5.2038	H-14→L+18 (15.6), H-14→L+21 (12.8), H-10→L+26 (5.2), H-12→L+18 (5.1)
	5	5.2039	H-14→L+18 (13.5), H-14→L+21 (11.6), H-10→L+26 (5.6)
	6	5.2085	H→L+39 (11.2), H-16→L+17 (10.8), H→L+36 (9.2), H→L+34 (6.4), H→L+42 (5.8), H-1→L+39 (5.3)
	7	5.2189	H-8→L+28 (8.6), H-8→L+24 (8.5), H-10→L+24 (5.5)
	8	5.2334	H-1→L+39 (11.4), H-1→L+36 (8.3), H→L+39 (6.9), H-1→L+42 (6.4), H-1→L+34 (6.3), H→L+36 (5.6)
	9	5.2395	H-18→L+10 (31.3), H-18→L+14 (9.1), H-18→L+12 (7.8)
	10	5.2436	H-2→L+34 (9.0), H-3→L+34 (7.3), H-2→L+40 (6.9), H-3→L+40 (6.1)
	11	5.2475	H-7→L+32 (9.9), H-5→L+29 (6.4), H-7→L+35 (5.4)
	12	5.2568	H-5→L+32 (10.4), H-7→L+29 (6.9), H-5→L+35 (5.4)
	13	5.2584	H-23→L+1 (78.7), H-23→L+2 (5.9)
	14	5.2606	H-22→L+4 (32.9), H-22→L+3 (26.7), H-22→L+2 (8.5), H-22→L+7 (6.2), H-22→L+6 (5.7), H-22→L+8 (5.7), H-22→L (5.1)
	15	5.2716	H-21→L+5 (58.2), H-21→L+7 (10.5), H-21→L+2 (10.4)
	16	5.2850	H-12→L+26 (7.3), H-12→L+22 (7.2)
	17	5.3105	H-2→L+34 (10.3), H-3→L+34 (8.9), H-2→L+40 (7.5), H-3→L+40 (6.9)
	18	5.3347	H-20→L+9 (64.6), H-20→L+5 (7.9)
	19	5.3569	H-9→L+26 (12.6), H-9→L+22 (12.4), H-9→L+15 (9.4), H-9→L+21 (5.5), H-9→L+14 (5.3)
	20	5.3985	H-6→L+32 (11.8), H-4→L+29 (9.0), H-6→L+35 (6.6)
	21	5.4096	H-4→L+32 (13.1), H-6→L+29 (8.5), H-4→L+35 (7.5)
	22	5.4490	H-19→L+10 (31.9), H-19→L+14 (9.4), H-19→L+12 (8.7), H-19→L (5.6)
	23	5.4763	H-11→L+24 (13.4), H-15→L+26 (8.7), H-15→L+28 (7.1), H-11→L+8 (5.7)

	24	5.4914	H-15→L+24 (10.7), H-15→L+26 (7.5), H-15→L+8 (6.9), H-11→L+28 (6.7), H-11→L+26 (6.1)
	25	5.5305	H→L+1 (51.5), H→L (43.3)
	26	5.5927	H→L+1 (40.3), H→L (34.4), H→L+2 (16.3)
	27	5.6856	H-1→L (52.0), H-1→L+1 (40.0)
	28	5.7329	H-1→L+1 (51.4), H-1→L (24.3), H-1→L+2 (16.1)
	29	5.7719	H→L+3 (30.6), H→L+2 (21.3), H→L (15.4), H→L+4 (14.1)
	30	5.8134	H-24→L+20 (16.5), H-25→L+20 (11.3), H-24→L+18 (9.3), H-24→L+19 (7.6), H-25→L+19 (5.5), H-25→L+18 (5.4), H-24→L+25 (5.2)
	31	5.8188	H-24→L+18 (18.3), H-25→L+18 (17.6), H-24→L+21 (17.0), H-25→L+21 (14.4)
	32	5.8212	H-2→L+2 (21.3), H-2→L+9 (19.4), H-2→L (14.9), H-2→L+5 (11.5), H-2→L+6 (5.4)
	33	5.8224	H-27→L+17 (43.3), H-27→L+16 (8.7), H-26→L+17 (8.4), H-27→L+15 (6.2), H-25→L+17 (5.4)
	34	5.8424	H→L+3 (53.9), H→L+4 (7.7), H→L+2 (7.6), H→L+7 (5.6)
	35	5.8547	H-5→L (40.9), H-3→L (11.7), H-4→L+1 (10.1)
	36	5.8616	H-5→L+1 (26.9), H-4→L+1 (14.9), H-4→L (14.4), H-3→L (11.6), H-6→L+1 (9.0)
	37	5.8748	H-4→L (38.2), H-4→L+1 (12.3), H-5→L (10.5), H-3→L (7.4), H-5→L+1 (6.7)
	38	5.8918	H-2→L (17.9), H-4→L (13.9), H-3→L+2 (8.4), H-2→L+9 (6.8), H-2→L+2 (6.1), H-5→L (5.4)

Supplementary Table 8. TD-DFT predicted singlet and triplet excited state energies and transition configurations of selected tetramer 2 of **(R, R)-DAACH**.

Excited state	*n*-th	Energy (eV)	Transition configuration (%)
S_n	1	5.4053	H→L (89.7)
T_n	1	5.1859	H-14→L+17 (16.5), H-14→L+16 (9.2), H-2→L+35 (9.2), H-14→L+15 (8.0), H-14→L+12 (5.6), H-14→L+14 (5.6)
	2	5.1874	H-13→L+19 (20.9), H-13→L+16 (14.5), H-13→L+15 (10.9)
	3	5.1911	H-15→L+12 (46.4), H-11→L+12 (8.4), H-15→L+18 (6.1), H-10→L+12 (5.7)
	4	5.1956	H-12→L+15 (20.1), H-12→L+18 (17.2), H-12→L+17 (10.5), H-12→L+19 (8.5)
	5	5.2022	H-14→L+17 (11.5), H-2→L+35 (11.4), H-14→L+16 (6.5), H-14→L+15 (5.6), H-2→L+30 (5.4)
	6	5.2057	H-19→L+5 (25.7), H-19→L+7 (10.9), H-18→L+5 (6.2), H-19→L+3 (5.5)
	7	5.2086	H→L+34 (13.7), H→L+38 (8.4), H→L+39 (6.4), H-13→L+19 (5.3)
	8	5.2329	H-5→L+35 (22.8), H-5→L+30 (10.4), H-5→L+33 (10.3), H-5→L+40 (6.7)
	9	5.2358	H-4→L+34 (15.1), H-4→L+38 (9.5), H-4→L+39 (7.3)
	10	5.2527	H-23→L+1 (41.5), H-23→L (30.2), H-23→L+2 (12.0)
	11	5.2634	H-22→L+4 (22.8), H-7→L+27 (8.2), H-22→L+3 (7.9), H-7→L+28 (6.5), H-22→L+1 (5.3), H-7→L+30 (5.2), H-6→L+27 (5.0)
	12	5.2658	H-16→L+9 (46.4), H-16→L+10 (15.0)
	13	5.2686	H-22→L+4 (26.5), H-22→L+3 (9.1), H-7→L+27 (6.9), H-22→L+1 (6.3), H-7→L+28 (5.7), H-22→L+2 (5.5)
	14	5.2842	H-9→L+25 (11.6), H-9→L+22 (10.2), H-9→L+24 (8.5), H-9→L+27 (5.1)
	15	5.2909	H-1→L+37 (30.3), H-1→L+41 (11.3), H-1→L+39 (6.9), H-1→L+35 (6.0)
	16	5.2939	H-11→L+21 (15.7), H-11→L+11 (6.9), H-11→L+18 (6.9)
	17	5.3109	H-3→L+37 (11.1), H-21→L+6 (7.9), H-6→L+27 (6.6), H-6→L+28 (5.7)
	18	5.3134	H-3→L+37 (16.4), H-6→L+27 (7.4), H-3→L+41 (6.2), H-6→L+28 (6.1)

	19	5.3215	H-21→L+6 (34.1), H-21→L+7 (13.9), H-21→L+5 (9.4), H-21→L+2 (5.3)
	20	5.3427	H-17→L+13 (25.0), H-17→L+14 (24.0), H-17→L+16 (17.1), H-17→L+20 (7.0)
	21	5.3547	H-10→L+21 (18.7), H-10→L+11 (9.6), H-10→L+18 (8.7), H-10→L+12 (6.5), H-10→L+24 (5.4)
	22	5.3661	H-8→L+25 (13.2), H-8→L+22 (12.3), H-8→L+24 (9.4), H-8→L+15 (6.3), H-8→L+17 (5.6), H-8→L+27 (5.5)
	23	5.4029	H→L (86.4)
	24	5.4290	H-18→L+9 (43.5), H-18→L+10 (13.4), H-19→L+9 (10.5)
	25	5.4460	H-20→L+5 (33.3), H-2→L (17.3), H-20→L+7 (13.7), H-20→L (8.3), H-20→L+3 (7.7)
	26	5.4612	H-2→L (75.2), H-20→L+5 (7.6)
	27	5.5682	H-1→L (85.9)
	28	5.5840	H-4→L (70.3), H-3→L (15.3)
	29	5.6028	H-2→L+1 (82.8), H-2→L+3 (8.6)
	30	5.6326	H→L+1 (82.7), H→L+2 (6.9), H→L+4 (5.0)
	31	5.6351	H-5→L (92.6)
	32	5.6548	H-3→L (76.1), H-4→L (16.8)
	33	5.6772	H-1→L+1 (80.3), H-1→L (6.3), H-1→L+3 (5.4)
	34	5.7152	H→L+2 (37.4), H→L+3 (34.0), H→L+4 (12.3), H→L+1 (9.3)
	35	5.7456	H-1→L+2 (81.6), H-1→L+4 (6.7)
	36	5.7657	H-5→L+1 (86.4), H-5→L+3 (7.2)
	37	5.7705	H-3→L+1 (83.3)
	38	5.7841	H-1→L+4 (63.2), H-1→L+2 (5.6)

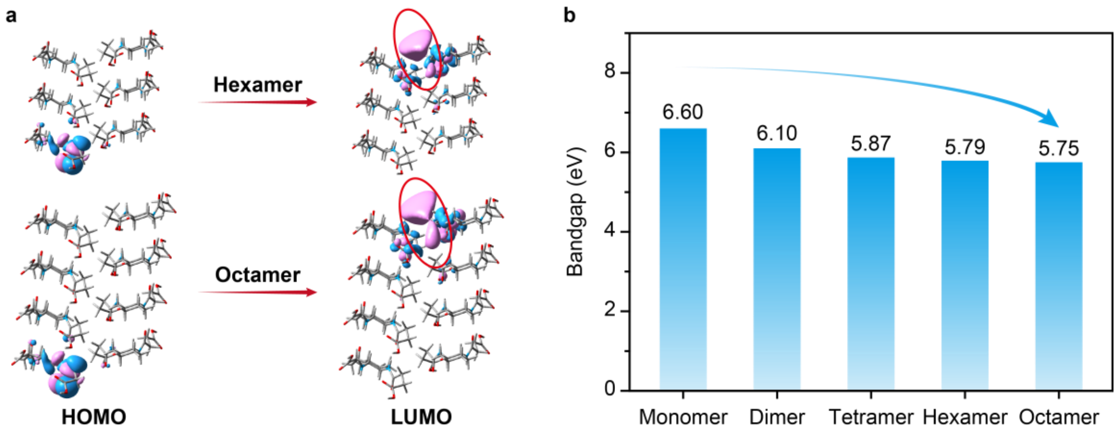

Supplementary Figure 37. (a) Frontier molecular orbital distributions for the selected hexamer and octamer and (b) calculated energy bandgaps for the selected aggregates extracted from **(R, R)-DAACH** single crystal.

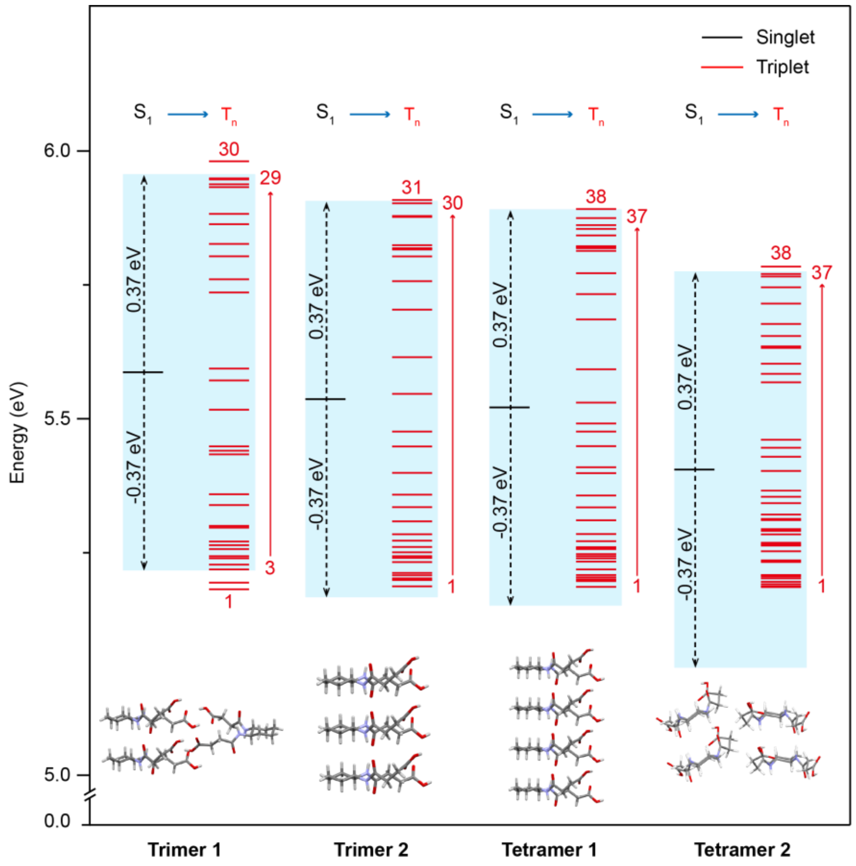

Supplementary Figure 38. TD-DFT calculated energy levels diagram based on the molecular structures of selected trimers and tetramer extracted from **(R, R)-DAACH** single crystal. The energy levels located in the blue background means the possible ISC channel for the generation of triplet excitons.

6. Design rationale verification of chiral clusterization strategy

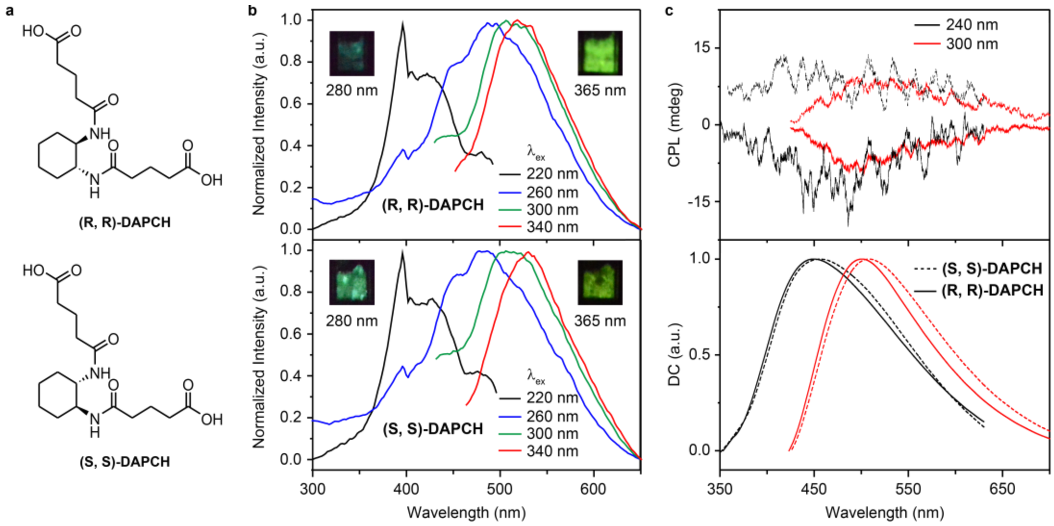

Supplementary Figure 39. (a) Chemical structures of **(R, R)/(S, S)-DAPCH**. (b) Afterglow spectra and (c) corresponding CPL properties of **(R, R)/(S, S)-DAPCH** powders upon excitation at different wavelength under ambient conditions.

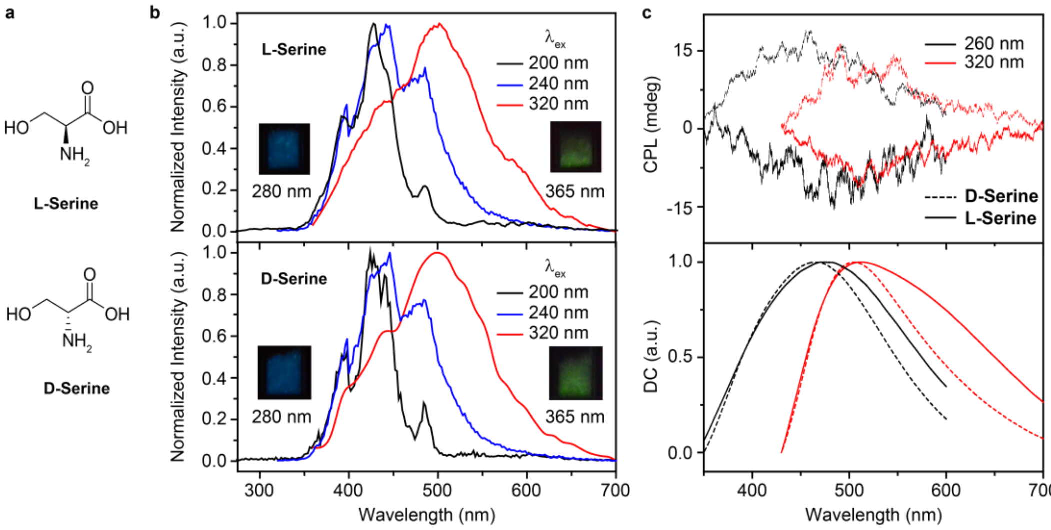

Supplementary Figure 40. (a) Chemical structures of **L/D-Serine**. (b) Afterglow spectra and (c) corresponding CPL properties of **L/D-Serine** powders upon excitation at different wavelength under ambient conditions.

Supplementary References

1. Ruiz-Carretero, A. et al. Stepwise self-assembly to improve solar cell morphology. *J. Mater. Chem. A.* **1**, 11674-11681 (2013).
2. Zhao, T. et al. Enhanced Circularly Polarized Luminescence from Reorganized Chiral Emitters on the Skeleton of a Zeolitic Imidazolate Framework. *Angew Chem Int Ed*. **58**, 4978-4982 (2019).
3. Han, J. et al. Enhanced Circularly Polarized Luminescence in Emissive Charge-Transfer Complexes. *Angew Chem Int Ed*. **58**, 7013-7019 (2019).
4. Tao, Y. et al. Resonance-Activated Spin-Flipping for Efficient Organic Ultralong Room-Temperature Phosphorescence. *Adv. Mater.* **30**, 1803856 (2018).
5. Hirata, S. & Vacha, M. Circularly Polarized Persistent Room-Temperature Phosphorescence from Metal-Free Chiral Aromatics in Air. *J Phys Chem Lett*. **7**, 1539-1545 (2016).
6. Liang, X. et al. Organic Room-Temperature Phosphorescence with Strong Circularly Polarized Luminescence Based on Paracyclophanes. *Angew Chem Int Ed*. **58**, 17220-17225 (2019).
7. Chen, W. et al. Long-Persistent Circularly Polarized Phosphorescence from Chiral Organic Ionic Crystals. *Chem.-Eur. J.* **24**, 17444-17448 (2018).
8. Li, H. et al. Stimuli‐Responsive Circularly Polarized Organic Ultralong Room Temperature Phosphorescence. *Angew Chem Int Ed*. **59**, 4756-4762 (2020).
9. Li, J. et al. Colour-tunable dual-mode afterglows and helical-array-induced mechanoluminescence from AIE enantiomers: Effects of molecular arrangement on formation and decay of excited states. *Chem. Eng. J.* **418**, 129167 (2021).
10. Wu, X. et al. Exploiting racemism enhanced organic room-temperature phosphorescence to demonstrate Wallach’s rule in the lighting chiral chromophores. *Nat. Commun.* **11**, 2145 (2020).
11. Yuan, J. et al. Direct population of triplet excited states through singlet–triplet transition for visible-light excitable organic afterglow. *Chem. Sci.* **10**, 5031-5038 (2019).
12. Li, H. et al. Fluorine-induced aggregate-interlocking for color-tunable organic afterglow with a simultaneously improved efficiency and lifetime. *Chem. Sci.* **12**, 3580-3586 (2021).
